# Peripheral instability gradient in macular thickness measurements across five optical coherence tomography systems: a prospective cross-sectional study of inter-device agreement and clinical monitoring risk

**DOI:** 10.1186/s12880-026-02480-3

**Published:** 2026-05-30

**Authors:** Fevzi Akkan, Ebru Gorgun

**Affiliations:** Department of Ophthalmology, Dunyagoz Eye Hospital, Nispetiye Cd. Yanarsu Sk. No:1, Etiler, Istanbul, Türkiye

**Keywords:** Macular thickness, Optical coherence tomography, Inter-device agreement, Peripheral instability gradient, ETDRS, Repeatability, Clinical decision-making

## Abstract

**Background:**

Cross-platform variability in optical coherence tomography (OCT) macular thickness measurements poses a clinically meaningful challenge for longitudinal patient assessment, particularly when patients are imaged on different devices over time. This study characterizes intra-device repeatability and inter-device agreement across five contemporary OCT systems, and introduces the Peripheral Instability Gradient (PIG)—the progressive deterioration in cross-device measurement concordance from the foveal center outward across ETDRS-defined macular zones.

**Methods:**

In this prospective, cross-sectional study, 38 healthy adults underwent macular imaging with five OCT devices: HRA Spectralis (Heidelberg Engineering), BMIZAR (TowardPi, 400 kHz swept-source OCTA), TOPCON Maestro 2, NIDEK RS-1 Glauvas, and HUVITZ HOCT-1/1F. Macular thickness was measured in the central 1-mm ETDRS subfield and the 3-mm and 6-mm inner rings (superior, nasal, inferior, and temporal sectors). Three consecutive scans were acquired per device during a single visit by a single experienced operator. Intra-device repeatability was assessed using ICC(3,1) and repeated-measures ANOVA with Bonferroni correction. Inter-device agreement was evaluated using ICC(2,1), Bland–Altman analysis, and absolute percentage error (APE). HRA-anchored comparisons were used for directional bias interpretation, and all 10 direct pairwise device comparisons were additionally performed as cross-validation.

**Results:**

All devices demonstrated excellent within-device repeatability (ICC > 0.85 across all regions). Inter-device agreement was strongly location-dependent, with PIG: ICC values declining from the central 1-mm subfield (HRA vs. HUVITZ: 0.942; HRA vs. NIDEK: 0.902) to the 6-mm ring (ICC < 0.40 for most pairs). This represents a relative decline of 40–67%. Central thickness ranged from 227.85 μm (TOPCON) to 290.73 μm (BMIZAR). Device-specific systematic biases were identified: BMIZAR overestimated HRA by a mean relative bias of 10.98%, and TOPCON underestimated HRA by a mean relative bias 13.65%, while HUVITZ (+ 2.60%) and NIDEK (+ 3.64%) showed smaller relative deviations (all biases as HRA minus comparator). Limits of agreement widened by a relative 30–50% from central to peripheral rings. Median APE was lowest for HUVITZ (2.39%) and highest for TOPCON (12.23%).

**Conclusions:**

Macular thickness measurements are highly repeatable within individual OCT systems but are not interchangeable across devices, with disagreement amplifying progressively toward peripheral macular zones. The Peripheral Instability Gradient demonstrates that cross-device follow-up carries the greatest risk in parafoveal and perifoveal monitoring. To ensure reliable longitudinal assessment, clinicians should maintain device consistency; when switching is unavoidable, device-specific baselines should be established and calibration adjustments applied.

**Supplementary Information:**

The online version contains supplementary material available at 10.1186/s12880-026-02480-3.

## Significance

**What is already known about this topic?**

• OCT macular thickness measurements show high within-device repeatability but differ substantially across platforms.

• Inter-device agreement tends to be higher in the central macular region than in peripheral zones.

• Direct interchangeability of macular thickness values across OCT platforms is not recommended.

**What does this study add?**

• First formal quantification of the Peripheral Instability Gradient (PIG)—a progressive, device-independent deterioration in inter-device agreement from the central 1-mm ETDRS subfield to the 6-mm perifoveal ring, with ICC declining by 40–67%.

• First comprehensive multi-device comparison to include a 400 kHz swept-source OCTA system (BMIZAR) alongside four SD-OCT platforms in a unified five-device framework.

• Evidence-based, zone-specific clinical recommendations for longitudinal monitoring and device-switching scenarios.

**Supplementary Information:**

The online version contains supplementary material available at 10.1186/s12880-026-02480-3.

## Introduction

Optical coherence tomography (OCT) has become indispensable in modern ophthalmic practice, enabling noninvasive, high-resolution visualization of retinal microstructure. Quantitative macular thickness assessment is central to the diagnosis and longitudinal monitoring of conditions such as diabetic macular edema (DME), age-related macular degeneration (AMD), and retinal vein occlusion (RVO)—conditions in which treatment decisions are frequently anchored to absolute thickness values and their change over time [1, 2].

However, a critical and underappreciated challenge persists: macular thickness measurements vary substantially across OCT platforms due to differences in optical design, scan acquisition protocols, and proprietary automated segmentation algorithms. These device-dependent variations can produce clinically meaningful discrepancies—differences that may rival treatment thresholds in conditions such as DME—making it essential to distinguish true pathological change from measurement artifact [2, 3]. While prior studies consistently demonstrate excellent within-device repeatability, inter-device comparability remains poorly characterized, particularly for newer technology platforms (e.g., swept-source OCT angiography devices) and for measurements extending beyond the central macular subfield [4, 5].

Previous investigations have compared OCT repeatability within single devices or evaluated agreement between two platforms, but comprehensive cross-platform comparisons involving five contemporary systems—spanning different generations (spectral-domain vs. swept-source) and clinical roles (research-grade, general-use)—are lacking. Critically, the spatial pattern of inter-device disagreement across ETDRS-defined macular zones has not been systematically quantified. Most studies report central subfield agreement without characterizing how agreement deteriorates toward parafoveal and perifoveal regions, where many clinically relevant pathologies manifest [6–9].

We hypothesized that inter-device agreement deteriorates progressively as the measurement region expands from the foveal center to peripheral macular zones. The Peripheral Instability Gradient (PIG) would reflect the cumulative effects of device-specific segmentation algorithm limitations, eye-tracking and fixation-decentering sensitivity, differences in scan density, and increasing retinal curvature and vessel shadowing in peripheral regions. Quantifying this gradient is essential, as it defines the spatial boundaries of reliable cross-device comparison and identifies which clinical monitoring scenarios carry the highest risk from device-related measurement noise [8, 10].

This prospective study evaluates intra-device repeatability and inter-device agreement of macular thickness measurements obtained from five OCT systems—HRA Spectralis, BMIZAR (a newer swept-source OCTA platform evaluated comprehensively for the first time in this context), TOPCON Maestro 2, NIDEK RS-1, and HUVITZ HOCT-1/1F—in healthy adults. The primary aim is to characterize the Peripheral Instability Gradient and its implications for longitudinal monitoring and clinical decision-making in the era of multi-platform OCT imaging.

## Materials and methods

### Study design and participants

This prospective, cross-sectional study was approved by the local institutional ethics committee (Approval number: E-10840098-202.3.02-165) and conducted in accordance with the Declaration of Helsinki. Written informed consent was obtained from all participants before enrollment.

Healthy adults aged 18–40 years were consecutively recruited from the outpatient ophthalmology clinic. Subjects with a history of ocular disease, previous ocular surgery, media opacities affecting image quality, systemic diseases known to affect the retina, poor fixation, or significant refractive error ( > ± 1.0 diopter spherical equivalent) were excluded. The refractive error threshold was selected to minimize confounding effects of axial-length–related retinal curvature and magnification differences, which can disproportionately affect peripheral ETDRS thickness measurements and automated segmentation performance. While the number of subjects was 38, the study design involved a comprehensive multi-sector analysis (9 sectors per eye) across 5 devices, resulting in over 3,400 discrete thickness measurements for analysis.

### Ophthalmic examination

All participants underwent a comprehensive ophthalmic examination, including best-corrected visual acuity assessment, intraocular pressure measurement, slit-lamp biomicroscopy, and dilated fundus examination. Only eyes with normal ophthalmic findings were included in the final analysis.

### OCT imaging protocol: devices and technical specifications

Five OCT systems were evaluated:

- **HRA Spectralis** (Heidelberg Engineering): Spectral-domain OCT combined with confocal scanning laser ophthalmoscopy. Active TruTrack eye-tracking enables precise scan registration, and retinal thickness is measured using device-specific automated segmentation algorithms.
- **BMIZAR** (TowardPi BMizar, 400 kHz): Ultra-widefield swept-source OCT angiography (SS-OCTA) platform. Swept-source technology employs a rapidly tunable laser source enabling deep tissue penetration and widefield imaging. Macular thickness and angiographic parameters are derived from repeated B-scan acquisition and motion-contrast analysis.
- **TOPCON Maestro 2**: Automated spectral-domain OCT integrated with a true-color fundus camera. Automated alignment, autofocus, and image capture generate ETDRS-based macular thickness maps from volumetric scans.
- **NIDEK RS-1 Glauvas**: Spectral-domain OCT utilizing high-speed A-scan acquisition and proprietary segmentation, with retinal thickness defined between the inner limiting membrane (ILM) and the retinal pigment epithelium (RPE).
- **HUVITZ HOCT-1/1F**: All-in-one spectral-domain OCT operating at approximately 840 nm, where depth-resolved retinal reflectivity is obtained via Fourier transformation of the interference spectrum.

### Acquisition protocol

Standardized macular cube/volumetric scans were acquired per manufacturer recommendations. To minimize operator- and time-related variability, all five devices were used during the same visit, on the same day, under identical lighting conditions, with scans acquired by a single experienced operator. Participants repositioned their head between three consecutive scans per device to simulate real-world clinical follow-up. Scans with poor image quality, motion artifacts, segmentation errors, or inadequate centration were excluded. Scans were accepted only when device-reported image quality indicators met or exceeded manufacturer-recommended minimum thresholds for clinical analysis. Automated proprietary segmentation was used without manual correction to reflect routine clinical practice.

### Measurement definition

Macular thickness was defined as the distance from the inner limiting membrane (ILM) to the RPE. Measurements were extracted from three ETDRS-defined regions: the central 1-mm subfield, the 3-mm inner ring (superior, nasal, inferior, temporal sectors), and the 6-mm inner ring (superior, nasal, inferior, temporal sectors). For the primary directional bias analyses, HRA Spectralis was used as the reference device for three reasons: (1) it is widely regarded as a research-grade benchmark in retinal imaging due to its active TruTrack eye-tracking system, high-density B-scan averaging, and regulatory approval for clinical trial use [7, 9]; (2) it has served as the reference standard in the majority of published OCT cross-platform agreement studies, enabling direct comparison of our findings with existing literature; and (3) its active eye-tracking provides superior scan registration accuracy, minimizing fixation-decentration artifacts that disproportionately affect peripheral ETDRS measurements. Importantly, designating HRA as the reference does not imply that HRA measurements are the ‘true’ ground truth; it establishes a consistent anchor for directional bias interpretation. To address inter-device comparability independent of a single reference device, we additionally performed direct all-pairwise cross-validation among the five OCT systems; all 10 device pairs were analyzed across each ETDRS region and are reported in Supplementary Table S1. By convention in this study, a positive HRA−comparator bias indicates that the comparator device underestimates relative to HRA, and a negative bias indicates overestimation. Readers should be aware that if HRA itself carries a systematic offset relative to histological or phantom ground truth, all reported biases would shift accordingly. However, because all four comparator devices were assessed simultaneously against the same HRA reference, any fixed HRA-specific offset would affect all bias estimates equally and in the same direction, leaving the relative ordering of inter-device biases, BMIZAR overestimating most, TOPCON underestimating most, and HUVITZ and NIDEK deviating least, mathematically invariant to reference choice.

### Repeatability assessment

Three consecutive macular scans (R1–R3 for the right eye; L1–L3 for the left eye) were acquired per device per eye during the same session. Right and left eyes were analyzed separately to account for potential interocular differences.

### Statistical Analysis

Continuous variables are presented as mean ± standard deviation (SD). A two-sided p-value < 0.05 was considered statistically significant. Within-device consistency (Intra-Device Repeatability) was evaluated using the intraclass correlation coefficient [ICC(3,1)], derived from a two-way mixed-effects model. Differences among three consecutive measurements (R1–R3 and L1–L3) were assessed using repeated-measures ANOVA with Bonferroni correction for multiple comparisons. No formal a priori sample size calculation was performed, consistent with the exploratory and agreement-characterization nature of this study. The sample size of *n* = 38 was determined based on feasibility considerations and is consistent with, and in several cases exceeds, the sample sizes employed in previously published cross-platform OCT agreement studies: Nam et al. (2024) evaluated four OCT devices in 21 subjects [9]; and Chalam et al. (2025) used *n* = 64 normal eyes of 32 participants [11]. Furthermore, the analytical framework of this study yields substantially more discrete measurements per subject than typical single-device repeatability studies. With 9 ETDRS sectors × 5 devices × 3 repetitions × 2 eyes, the study yielded over 8100 individual thickness measurements from 38 participants, providing a robust dataset for agreement quantification. ICC-based agreement analyses are primarily determined by the magnitude of between-subject variance relative to within-subject/device variance rather than by raw sample size alone; the consistently large between-subject standard deviations observed (14–25 μm across regions and devices) relative to within-device measurement noise ensure adequate statistical power for the primary ICC and Bland–Altman comparisons. Notwithstanding, the modest sample size limits the precision of ICC confidence intervals, as reflected in the wide 95% CIs reported throughout, and precludes reliable stratified subgroup analyses. This is explicitly acknowledged as a limitation of the study.

Primary inter-method reliability analyses were performed between HRA, used as the directional reference/anchor device, and each of the four comparator devices using ICC(2,1) (two-way random-effects model, absolute agreement, single measurements), with 95% confidence intervals and p-values reported. To avoid reliance on a single reference device, we also performed direct all-pairwise cross-validation among the five OCT systems. For this supplementary analysis, the three repeated scans per eye were averaged, and all 10 device pairs were compared at the eye level across the central 1-mm subfield and all 3-mm and 6-mm ETDRS sectors using ICC(2,1), Bland–Altman bias, 95% limits of agreement, and LoA width. ICC values were interpreted according to established thresholds: poor (< 0.50), moderate (0.50–0.75), good (0.75–0.90), and excellent (> 0.90) [12]. A negative HRA−comparator bias indicates that the comparator device overestimates relative to HRA, while a positive bias indicates that the comparator device underestimates relative to HRA.

Bland–Altman analysis was performed to assess method agreement. Mean bias was expressed as a percentage difference relative to HRA, with 95% limits of agreement (LoA). LoA width (upper minus lower limit) served as the primary indicator of measurement dispersion. Proportional bias was assessed via linear regression of inter-method differences against measurement means; a statistically significant regression slope indicated the presence of proportional bias (*p* < 0.05) [13]. Interocular differences within each device were assessed using paired-samples t-tests with bootstrap resampling. Absolute Percentage Error (APE) was calculated as [|measured value − HRA reference| / HRA reference] × 100%. Median APE with 95% CI and the 95th percentile were reported to capture both central tendency and extreme discrepancies.

To quantify the Peripheral Instability Gradient (PIG), agreement metrics were systematically compared across the three ETDRS ring sizes (1 mm, 3 mm, 6 mm) for each device pair. The PIG is formally defined as a composite, spatially-structured deterioration in inter-device measurement concordance from the central 1 mm ETDRS subfield to the 6 mm perifoveal ring, operationalized through three complementary agreement metrics assessed in parallel: (1) relative ICC(2,1) decline; the percentage decrease in ICC from the 1 mm to the 6 mm zone, serving as the primary indicator of loss in cross-device reliability; (2) relative LoA width expansion; the percentage increase in Bland–Altman 95% limits of agreement width from the 1 mm to the 6 mm zone, serving as the primary indicator of increased measurement dispersion; and (3) absolute percentage error (APE) escalation; the increase in median APE from central to peripheral zones, capturing the magnitude of clinically relevant measurement discrepancy. The PIG is considered present and quantified when all three metrics demonstrate a monotonic deterioration (worsening) from the 1 mm to the 6 mm zone. The overall PIG magnitude for a given device pair is summarized by the relative ICC decline (primary metric), with LoA expansion and APE escalation serving as corroborating indicators. This composite definition ensures that the PIG reflects true, multidimensional deterioration in cross-device agreement rather than any single metric in isolation. All analyses were performed using IBM SPSS Statistics v26 (IBM Corporation, Armonk, NY, USA) and MedCalc v14 (MedCalc Software, Ostend, Belgium).

## Results

A total of 38 healthy adults (76 eyes; mean age 26.6 ± 3.4 years, median 26 years, range 21–35; 26 females [68.4%], 12 males [31.6%]) completed the study. All participants had normal findings on comprehensive ophthalmic examination and were included in the final analysis.

The main finding of this study is the Peripheral Instability Gradient (PIG): inter-device agreement, quantified by ICC(2,1), Bland–Altman limits of agreement (LoA), and absolute percentage error (APE), deteriorated systematically and progressively as the measurement region expanded from the central 1-mm ETDRS subfield to the 3-mm and 6-mm inner macular rings. This gradient was consistent across all device pairs. Within-device repeatability was consistently high (ICC(3,1) > 0.85) across all regions and devices. The following subsections present these findings by ETDRS region, followed by a dedicated summary of intra-device repeatability.

### Central macular thickness (1-mm ETDRS subfield)

Central macular thickness differed significantly among the five devices (repeated-measures ANOVA, F = 156.3, *p* < 0.001). When the mean of both eyes was considered, measurements were: HRA 258.62 ± 22.79 μm, BMIZAR 290.73 ± 24.95 μm (+ 12.4% relative to HRA), HUVITZ 252.06 ± 21.79 μm (− 2.5% relative to HRA), NIDEK 249.66 ± 22.36 μm (− 3.5% relative to HRA), and TOPCON 227.85 ± 22.63 μm (− 11.9% relative to HRA). All pairwise comparisons with HRA were statistically significant (all *p* < 0.001). Right–left eye symmetry was preserved across all devices for the central subfield (all *p* > 0.23; Table 1).

**Table 1:** Comparison of central (1-mm) and Inner (3-mm) ETDRS macular thickness measurements across five devices

(*n* = 38)	I	II	III	IV	V	*p* Value ᵃ	Pairwise Comparisons			
	HRA	BMIZAR	HUVITZ	NIDEK	TOPCON					
	Mean (SD.)	Mean (SD.)	Mean (SD.)	Mean (SD.)	Mean (SD.)		I vs. II	I vs. III	I vs. IV	I vs. V
**Central Macular Thickness (1-mm) (µm)**										
Mean of Right and Left Eyes (µm)	258.62 (22.79)	290.73 (24.95)	252.06 (21.79)	249.66 (22.36)	227.85 (22.63)	< 0.001	< 0.001	< 0.001	< 0.001	< 0.001
Right Eye Mean (Rep 1–3) (µm)	258.31 (23.20)	289.02 (23.52)	251.54 (22.15)	250.17 (23.51)	227.38 (22.96)	< 0.001	< 0.001	< 0.001	< 0.001	< 0.001
Left Eye Mean (Rep 1–3) (µm)	258.93 (22.52)	292.44 (29.05)	252.59 (21.80)	249.15 (21.94)	228.32 (22.53)	< 0.001	< 0.001	< 0.001	< 0.001	< 0.001
** P value (Right vs. Left) **ᵗ	0.281	0.274	0.283	0.516	0.230					
**Inner Macular Thickness (3-mm) (µm)**										
** Superior**										
Mean of Right and Left Eyes (µm)	346.22 (17.33)	370.36 (18.04)	322.19 (16.40)	333.98 (16.34)	314.25 (15.68)	< 0.001	< 0.001	< 0.001	< 0.001	< 0.001
Right Eye Mean (Rep 1–3) (µm)	346.03 (18.34)	370.61 (17.21)	322.07 (17.67)	334.39 (16.53)	312.70 (19.74)	< 0.001	< 0.001	< 0.001	< 0.001	< 0.001
Left Eye Mean (Rep 1–3) (µm)	346.42 (16.88)	370.11 (20.49)	322.32 (16.28)	333.57 (16.38)	315.81 (16.77)	< 0.001	< 0.001	< 0.001	< 0.001	< 0.001
** P value (Right vs. Left) **ᵗ	0.715	0.829	0.869	0.187	0.407					
** Nasal**										
Mean of Right and Left Eyes (µm)	343.93 (19.09)	371.35 (19.45)	321.79 (16.72)	331.46 (18.38)	312.55 (18.22)	< 0.001	< 0.001	< 0.001	< 0.001	< 0.001
Right Eye Mean (Rep 1–3) (µm)	343.77 (18.88)	370.62 (18.28)	320.31 (15.92)	331.08 (18.41)	311.96 (18.29)	< 0.001	< 0.001	< 0.001	< 0.001	< 0.001
Left Eye Mean (Rep 1–3) (µm)	344.09 (19.50)	372.08 (22.31)	323.28 (18.28)	331.85 (18.49)	313.14 (18.66)	< 0.001	< 0.001	< 0.001	< 0.001	< 0.001
** P value (Right vs. Left) **ᵗ	0.635	0.487	**0.021**	0.166	0.267					
** Inferior**										
Mean of Right and Left Eyes (µm)	343.71 (17.19)	369.13 (18.04)	319.17 (15.59)	330.29 (15.88)	312.96 (16.50)	< 0.001	< 0.001	< 0.001	< 0.001	< 0.001
Right Eye Mean (Rep 1–3) (µm)	343.00 (16.87)	368.15 (16.63)	318.21 (15.34)	329.63 (15.61)	312.64 (16.65)	< 0.001	< 0.001	< 0.001	< 0.001	< 0.001
Left Eye Mean (Rep 1–3) (µm)	344.43 (17.72)	370.11 (20.75)	320.12 (16.35)	330.95 (17.00)	313.28 (16.73)	< 0.001	< 0.001	< 0.001	< 0.001	< 0.001
** P value (Right vs. Left) **ᵗ	**0.029**	0.342	0.051	0.326	0.421					
** Temporal**										
Mean of Right and Left Eyes (µm)	331.05 (17.43)	357.53 (19.63)	309.68 (15.68)	317.13 (16.61)	300.89 (16.21)	< 0.001	< 0.001	< 0.001	< 0.001	< 0.001
Right Eye Mean (Rep 1–3) (µm)	331.22 (17.32)	356.54 (17.43)	309.43 (15.53)	317.41 (16.17)	300.80 (16.47)	< 0.001	< 0.001	< 0.001	< 0.001	< 0.001
Left Eye Mean (Rep 1–3) (µm)	330.88 (17.69)	358.51 (23.67)	309.94 (16.22)	316.85 (17.19)	300.99 (16.43)	< 0.001	< 0.001	< 0.001	< 0.001	< 0.001
** P value (Right vs. Left) **ᵗ	0.518	0.460	0.534	0.275	0.841					

ᵃ Repeated measures analysis of variance (ANOVA) was performed to evaluate the differences in measurement results between five devices in the same patients, Adjustment for multiple comparisons: Bonferroni, ᵗ Paired Samples t-test (Bootsrap), SD. Standard Deviation, R1–R3 and L1–L3 represent three repeated measurements obtained from the right and left eyes, respectively

The central subfield yielded the highest inter-device agreement of all ETDRS regions, consistent with the PIG. HRA–HUVITZ demonstrated excellent agreement (ICC = 0.942; 95% CI: 0.207–0.985; *p* < 0.001) and HRA–NIDEK similarly excellent agreement (ICC = 0.902; 95% CI: 0.051–0.975; *p* < 0.001). In contrast, HRA–BMIZAR (ICC = 0.485; 95% CI: −0.037–0.827; *p* < 0.001) and HRA–TOPCON (ICC = 0.511; 95% CI: −0.010–0.848; *p* < 0.001) were classified as moderate. This pattern was consistent when the right and left eyes were analyzed separately (Table 2).

Bland–Altman analysis revealed distinct device-specific systematic biases at the central subfield. BMIZAR demonstrated a pronounced negative bias in the HRA−comparator difference (HRA vs. BMIZAR mean bias: −10.98%; 95% CI: −11.58 to − 10.39%; LoA width 12.6%; proportional bias *p* = 0.010), indicating that BMIZAR overestimates macular thickness relative to HRA by approximately 10.98%; this overestimation worsens with increasing retinal thickness. TOPCON exhibited the largest positive bias in the HRA−comparator difference (HRA vs. TOPCON mean bias: +13.65%; 95% CI: 13.19–14.11%; LoA width 9.73%; proportional bias *p* < 0.001), indicating that TOPCON underestimates macular thickness relative to HRA by approximately 13.65%. HUVITZ (mean bias + 2.60%; 95% CI: 2.26–2.94%; LoA width 7.15%; proportional bias *p* = 0.042) and NIDEK (mean bias + 3.64%; 95% CI: 3.19–4.08%; LoA width 9.39%; proportional bias *p* = 0.682) showed substantially lower central bias. APE analyses were consistent with these findings: median APE was lowest for HUVITZ (2.39%; 95% CI: 2.25–2.83%) and NIDEK (3.69%; 95% CI: 3.49–3.93%), and highest for TOPCON (12.23%; 95% CI: 11.72–12.79%) and BMIZAR (11.46%; 95% CI: 10.90–12.18%) (Table 3) [13].

### Inner macular thickness: 3-mm ETDRS ring

Significant inter-device differences were present in all four sectors of the 3-mm ring (all *p* < 0.001; Table 1). In the **superior sector**, mean values were: HRA 346.22 ± 17.33 μm, BMIZAR 370.36 ± 18.04 μm, HUVITZ 322.19 ± 16.40 μm, NIDEK 333.98 ± 16.34 μm, and TOPCON 314.25 ± 15.68 μm. In the **nasal sector**, values were 343.93 ± 19.09, 371.35 ± 19.45, 321.79 ± 16.72, 331.46 ± 18.38, and 312.55 ± 18.22 μm, respectively; a significant right–left asymmetry was noted for HUVITZ alone (*p* = 0.021). In the **inferior sector** (343.71, 369.13, 319.17, 330.29, and 312.96 μm), a significant interocular difference was detected only for HRA (*p* = 0.029). In the **temporal sector** (331.05, 357.53, 309.68, 317.13, and 300.89 μm), no significant right–left differences were observed for any device (all *p* > 0.46). Isolated interocular asymmetries were confined to specific device–sector combinations without a consistent lateralization pattern across devices.

The PIG became clearly manifest at the 3-mm ring: ICC values declined substantially compared with the central subfield across all device pairs. HRA–NIDEK retained the highest agreement, with moderate-to-good ICC values across all sectors (superior: 0.714; nasal: 0.759; inferior: 0.673; temporal: 0.703; all *p* < 0.001). HRA–BMIZAR and HRA–HUVITZ comparisons fell predominantly in the low-to-moderate range (ICC 0.43–0.54), while HRA–TOPCON exhibited the weakest agreement across all sectors, with ICC values ranging from 0.27 to 0.39—classified as poor-to-moderate. These patterns were replicated in right- and left-eye analyses separately (Table 2) [12].

Systematic biases persisted across all 3-mm sectors. In the **superior sector**, BMIZAR demonstrated a negative bias of − 6.49% (LoA width 9.58%; proportional bias *p* = 0.004; median APE 6.98%), while TOPCON showed a positive bias of + 10.32% (LoA width 20.98%; proportional bias *p* = 0.005; median APE 8.94%). HUVITZ exhibited a positive bias of + 7.49% (LoA width 8.18%; APE 6.90%), and NIDEK had the smallest bias and narrowest LoA (bias + 3.69%; LoA width 9.24%; APE 3.80%). In the **nasal** and **inferior** sectors, BMIZAR maintained consistent negative biases (− 7.35% and − 6.86%, respectively), while HUVITZ and TOPCON showed positive biases in the range of + 7–10%. NIDEK continued to demonstrate the most favorable agreement profile in each sector with median APE values of 3.94% (nasal) and 4.08% (inferior). In the **temporal sector**, BMIZAR bias was − 7.35% (LoA 9.96%; APE 7.66%) and TOPCON bias was + 10.05% (LoA 8.32%; APE 9.00%), while NIDEK remained the closest comparator (bias + 4.41%; APE 4.24%). Proportional bias was statistically significant in several device–sector combinations across the 3-mm ring, reflecting non-uniform measurement model differences that vary with absolute retinal thickness [12, 13] (Table 3).

**Table 2:** Inter-device reliability of ETDRS macular thickness measurements (ICC analysis)

	HRA vs. BMIZAR		HRA vs. HUVITZ		HRA vs. NIDEK		HRA vs. TOPCON	
	ICC (95% C.I.)	*p*	ICC (95% C.I.)	*p*	ICC (95% C.I.)	*p*	ICC (95% C.I.)	*p*
**Central Macular Thickness (1-mm ETDRS) (µm)**								
R & L Means	0.485 (-0.037/0.827)	< 0.001	0.942 (0.207/0.985)	< 0.001	0.902 (0.051/0.975)	< 0.001	0.511 (-0.010/0.848)	< 0.001
Means of R	0.512 (-0.023/0.846)	< 0.001	0.931 (0.357/0.980)	< 0.001	0.888 (0.412/0.963)	< 0.001	0.515 (-0.011/0.850)	< 0.001
Means of L	0.440 (-0.084/0.782)	< 0.001	0.943 (0.258/0.985)	< 0.001	0.897 (-0.020/0.977)	< 0.001	0.508 (-0.011/0.846)	< 0.001
**Inner Macular Thickness (3-mm) (µm)**								
** Superior**								
R & L Means	0.465 (-0.047/0.812)	< 0.001	0.477 (-0.018/0.828)	< 0.001	0.714 (-0.068/0.913)	< 0.001	0.273 (-0.050/0.650)	< 0.001
Means of R	0.459 (-0.046/0.809)	< 0.001	0.465 (-0.058/0.809)	< 0.001	0.714 (-0.020/0.904)	< 0.001	0.200 (-0.091/0.519)	< 0.001
Means of L	0.448 (-0.086/0.786)	< 0.001	0.479 (-0.006/0.832)	< 0.001	0.701 (-0.075/0.911)	< 0.001	0.331 (-0.033/0.717)	< 0.001
** Nasal**								
R & L Means	0.436 (-0.051/0.793)	< 0.001	0.544 (-0.024/0.861)	< 0.001	0.759 (-0.060/0.931)	< 0.001	0.392 (-0.017/0.773)	< 0.001
Means of R	0.452 (-0.032/0.809)	< 0.001	0.475 (-0.045/0.819)	< 0.001	0.752 (-0.063/0.929)	< 0.001	0.387 (-0.014/0.770)	< 0.001
Means of L	0.407 (-0.089/0.755)	< 0.001	0.605 (-0.020/0.889)	< 0.001	0.764 (-0.051/0.931)	< 0.001	0.395 (-0.029/0.771)	< 0.001
** Inferior**								
R & L Means	0.444 (-0.039/0.802)	< 0.001	0.455 (-0.014/0.816)	< 0.001	0.673 (-0.079/0.899)	< 0.001	0.358 (-0.012/0.748)	< 0.001
Means of R	0.438 (-0.028/0.801)	< 0.001	0.438 (-0.017/0.805)	< 0.001	0.637 (-0.079/0.877)	< 0.001	0.358 (-0.015/0.747)	< 0.001
Means of L	0.436 (-0.076/0.783)	< 0.001	0.475 (-0.019/0.826)	< 0.001	0.694 (-0.076/0.907)	< 0.001	0.359 (-0.016/0.748)	< 0.001
** Temporal**								
R & L Means	0.431 (-0.054/0.788)	< 0.001	0.529 (-0.017/0.855)	< 0.001	0.703 (-0.063/0.918)	< 0.001	0.362 (-0.016/0.750)	< 0.001
Means of R	0.463 (-0.019/0.819)	< 0.001	0.511 (-0.020/0.846)	< 0.001	0.698 (-0.063/0.916)	< 0.001	0.359 (-0.017/0.748)	< 0.001
Means of L	0.383 (-0.098/0.728)	< 0.001	0.542 (-0.026/0.860)	< 0.001	0.707 (-0.065/0.918)	< 0.001	0.364 (-0.023/0.750)	< 0.001
**Inner Macular Thickness (6-mm) (µm)**								
** Superior**								
R & L Means	0.384 (-0.010/0.769)	< 0.001	0.341 (-0.011/0.735)	< 0.001	0.492 (-0.081/0.816)	< 0.001	0.349 (-0.009/0.742)	< 0.001
Means of R	0.371 (-0.010/0.760)	< 0.001	0.330 (-0.018/0.722)	< 0.001	0.528 (-0.076/0.838)	< 0.001	0.341 (-0.011/0.734)	< 0.001
Means of L	0.400 (-0.019/0.778)	< 0.001	0.347 (-0.027/0.734)	< 0.001	0.461 (-0.088/0.794)	< 0.001	0.361 (-0.012/0.751)	< 0.001
** Nasal**								
R & L Means	0.386 (-0.019/0.768)	< 0.001	0.415 (-0.058/0.776)	< 0.001	0.655 (-0.068/0.882)	< 0.001	0.343 (-0.023/0.732)	< 0.001
Means of R	0.377 (-0.026/0.758)	< 0.001	0.422 (-0.044/0.785)	< 0.001	0.635 (-0.061/0.869)	< 0.001	0.340 (-0.033/0.725)	< 0.001
Means of L	0.388 (-0.031/0.765)	< 0.001	0.364 (-0.104/0.698)	< 0.001	0.668 (-0.064/0.888)	< 0.001	0.335 (-0.042/0.718)	< 0.001
** Inferior**								
R & L Means	0.321 (-0.029/0.710)	< 0.001	0.298 (-0.014/0.693)	< 0.001	0.492 (-0.098/0.803)	< 0.001	0.342 (-0.007/0.737)	< 0.001
Means of R	0.325 (-0.022/0.716)	< 0.001	0.278 (-0.029/0.665)	< 0.001	0.427 (-0.102/0.748)	< 0.001	0.327 (-0.014/0.720)	< 0.001
Means of L	0.309 (-0.069/0.679)	< 0.001	0.331 (-0.017/0.723)	< 0.001	0.526 (-0.094/0.816)	< 0.001	0.369 (-0.012/0.757)	< 0.001
** Temporal**								
R & L Means	0.451 (-0.041/0.805)	< 0.001	0.275 (-0.020/0.666)	< 0.001	0.396 (-0.077/0.753)	< 0.001	0.340 (-0.021/0.730)	< 0.001
Means of R	0.431 (-0.038/0.794)	< 0.001	0.238 (-0.043/0.610)	< 0.001	0.370 (-0.080/0.730)	< 0.001	0.322 (-0.029/0.710)	< 0.001
Means of L	0.452 (-0.075/0.794)	< 0.001	0.289 (-0.041/0.672)	< 0.001	0.416 (-0.083/0.765)	< 0.001	0.344 (-0.050/0.722)	< 0.001

Inter-method reliability was evaluated using the intraclass correlation coefficient (ICC), two-way random-effects model with absolute agreement [ICC(2,1)]

**Table 3:** Bland–Altman agreement analysis, proportional bias, and absolute percentage error for inter-device comparison of central and inner macular thickness measurements (1-mm, 3-mm, and 6-mm ETDRS rings), using HRA as the reference method

	Mean bias % (95% CI)	LoA width	Proportional bias (*p*)	Median APE % (95% CI)
**Central Macular Thickness (1-mm ETDRS) (µm)**				
HRA vs. BMIZAR	−10.98 (− 11.58 to − 10.39)	12.6	0.010	11.46 (10.90–12.18)
HRA vs. HUVITZ	2.60 (2.26–2.94)	7.15	0.042	2.39 (2.25–2.83)
HRA vs. NIDEK	3.64 (3.19–4.08)	9.39	0.682	3.69 (3.49–3.93)
HRA vs. TOPCON	13.65 (13.19–14.11)	9.73	< 0.001	12.23 (11.72–12.79)
**Inner Macular Thickness (3-mm ETDRS) (µm)**				
** Superior**				
HRA vs. BMIZAR	−6.49 (− 6.94 to − 6.03)	9.58	0.004	6.98 (6.45–7.35)
HRA vs. HUVITZ	7.49 (7.10–7.87)	8.18	0.359	6.90 (6.68–7.10)
HRA vs. NIDEK	3.69 (3.25–4.12)	9.24	0.003	3.80 (3.66–4.12)
HRA vs. TOPCON	10.32 (9.33–11.31)	20.98	0.005	8.94 (8.34–9.64)
** Nasal**				
HRA vs. BMIZAR	−7.35 (− 7.85 to − 6.86)	10.42	< 0.001	8.11 (7.72–8.47)
HRA vs. HUVITZ	6.88 (6.54–7.22)	7.17	0.001	6.25 (5.98–6.49)
HRA vs. NIDEK	3.79 (3.37–4.20)	8.76	0.045	3.94 (3.71–4.08)
HRA vs. TOPCON	10.08 (9.66–10.50)	8.86	0.850	9.19 (8.77–9.69)
** Inferior**				
HRA vs. BMIZAR	−6.86 (− 7.26 to − 6.46)	8.42	0.008	7.39 (6.96–7.86)
HRA vs. HUVITZ	7.70 (7.43–7.97)	5.74	0.055	7.26 (6.98–7.49)
HRA vs. NIDEK	4.09 (3.63–4.54)	9.6	0.003	4.08 (3.92–4.26)
HRA vs. TOPCON	9.86 (9.51–10.20)	7.29	0.816	8.97 (8.59–9.35)
** Temporal**				
HRA vs. BMIZAR	−7.35 (− 7.82 to − 6.88)	9.96	0.054	7.66 (7.17–8.08)
HRA vs. HUVITZ	6.90 (6.63–7.17)	5.74	0.008	6.36 (6.07–6.80)
HRA vs. NIDEK	4.41 (4.05–4.77)	7.62	0.049	4.24 (4.06–4.55)
HRA vs. TOPCON	10.05 (9.66–10.45)	8.32	0.428	9.00 (8.57–9.62)
**Inner Macular Thickness (6-mm ETDRS) (µm)**				
** Superior**				
HRA vs. BMIZAR	−7.81 (− 8.05 to − 7.57)	5.12	< 0.001	8.66 (8.33–8.85)
HRA vs. HUVITZ	9.87 (9.50–10.25)	7.95	0.294	8.96 (8.57–9.25)
HRA vs. NIDEK	5.88 (5.37–6.39)	10.81	< 0.001	5.92 (5.44–6.25)
HRA vs. TOPCON	9.92 (9.62–10.22)	6.29	0.916	8.87 (8.55–9.47)
** Nasal**				
HRA vs. BMIZAR	−8.00 (− 8.33 to − 7.67)	6.98	0.020	8.71 (8.26–9.12)
HRA vs. HUVITZ	8.39 (7.38–9.40)	21.27	0.936	7.03 (6.75–7.33)
HRA vs. NIDEK	4.02 (3.50–4.53)	10.88	0.006	4.27 (3.87–4.59)
HRA vs. TOPCON	9.78 (9.23–10.32)	11.53	0.244	8.44 (8.27–8.68)
** Inferior**				
HRA vs. BMIZAR	−8.78 (− 9.20 to − 8.36)	8.88	0.050	9.62 (9.06–9.94)
HRA vs. HUVITZ	10.81 (10.37–11.25)	9.34	0.707	10.26 (9.69–10.51)
HRA vs. NIDEK	5.74 (5.07–6.42)	14.28	0.018	6.10 (5.64–6.40)
HRA vs. TOPCON	10.12 (9.81–10.44)	6.68	0.210	9.19 (8.84–9.58)
** Temporal**				
HRA vs. BMIZAR	−7.04 (− 7.46 to − 6.62)	8.92	0.012	7.75 (7.35–8.43)
HRA vs. HUVITZ	12.09 (11.45–12.73)	13.44	0.224	11.46 (11.01–11.80)
HRA vs. NIDEK	7.39 (6.75–8.02)	13.38	< 0.001	7.01 (6.76–7.53)
HRA vs. TOPCON	10.46 (9.92–11.00)	11.45	0.621	9.82 (9.38–10.18)

Agreement between methods was evaluated using Bland–Altman analysis, linear regression for proportional bias, and absolute percentage error (APE) analysis

Mean bias is expressed as percentage difference relative to the reference method, LoA width represents the difference between the upper and lower limits of agreement derived from Bland–Altman analysis, Proportional bias p-values were obtained from regression slope estimates, APE indicates absolute percentage error

### Inner macular thickness: 6-mm ETDRS ring

All sectors of the 6-mm ring demonstrated significant inter-device differences (all *p* < 0.001; Table 4). In the **superior sector**, mean values were: HRA 306.00 ± 15.07 μm, BMIZAR 331.89 ± 14.70 μm, HUVITZ 278.58 ± 13.81 μm, NIDEK 289.07 ± 13.33 μm, and TOPCON 278.45 ± 14.27 μm. In the **nasal sector** (HRA: 320.20 ± 15.91 μm; BMIZAR: 348.11 ± 16.96; HUVITZ: 295.99 ± 17.38; NIDEK: 307.95 ± 15.45; TOPCON: 291.83 ± 15.16), significant right–left eye differences were detected for HRA (*p* = 0.027), BMIZAR (*p* = 0.019), and NIDEK (*p* = 0.015), but not for HUVITZ (*p* = 0.680) or TOPCON (*p* = 0.938). In the **inferior sector** (HRA: 295.46 ± 14.32; BMIZAR: 324.04 ± 16.07; HUVITZ: 266.75 ± 13.45; NIDEK: 279.65 ± 14.68; TOPCON: 268.38 ± 13.90 μm), no significant interocular differences were observed (all *p* > 0.22). In the **temporal sector** (HRA: 290.14 ± 15.04; BMIZAR: 312.23 ± 16.07; HUVITZ: 259.05 ± 14.05; NIDEK: 270.27 ± 12.88; TOPCON: 262.84 ± 14.47 μm), all pairwise comparisons with HRA were significant (all *p* < 0.001), with no right–left differences (all *p* > 0.25).

The 6-mm ring represented the nadir of inter-device agreement, demonstrating the full magnitude of the PIG. HRA–NIDEK, the strongest-performing pair, fell to low-to-moderate agreement across all sectors (superior: 0.492; nasal: 0.655; inferior: 0.492; temporal: 0.396). Agreement for HRA–BMIZAR, HRA–HUVITZ, and HRA–TOPCON was predominantly poor across all 6-mm sectors (most ICC values < 0.40), representing a relative ICC decline of 40–67% compared with the central subfield. These results were consistent for both mean-of-eyes and lateralized analyses (Table 2).

Inter-device differences were most pronounced in the 6-mm ring, with both LoA widths and APE values exceeding those of all smaller ETDRS regions. In the **superior sector**, BMIZAR showed a negative bias of − 7.81% (LoA width 5.12%; significant proportional bias *p* < 0.001; APE 8.66%), while HUVITZ (+ 9.87%; LoA 7.95%; APE 8.96%) and TOPCON (+ 9.92%; LoA 6.29%; APE 8.87%) demonstrated high positive biases. NIDEK maintained the most favorable profile (bias + 5.88%; LoA 10.81%; APE 5.92%). In the **temporal sector**—which exhibited the widest LoA values of the entire study—HUVITZ demonstrated a bias of + 12.09% with a LoA width of 13.44% and APE of 11.46%, while NIDEK reached its highest APE value at this location (bias + 7.39%; LoA 13.38%; APE 7.01%). BMIZAR’s temporal 6-mm bias was − 7.04% (LoA 8.92%; APE 7.75%) and TOPCON’s was + 10.46% (LoA 11.45%; APE 9.82%). Across the **nasal** and **inferior** 6-mm sectors, HUVITZ LoA widths reached their highest values (nasal: 21.27%), reflecting substantial measurement dispersion at peripheral macular zones. Proportional bias was significant in several 6-mm comparisons, particularly for BMIZAR and NIDEK, indicating thickness-dependent disagreement in peripheral regions [13] (Table 3).

### Peripheral instability gradient: quantitative summary

To explicitly quantify the PIG, ICC(2,1) values, and LoA widths were compared systematically across the three ETDRS ring sizes for each device pair (Tables 2 and 3). For the best-performing pair (HRA–HUVITZ), ICC declined from 0.942 at the central subfield to 0.477 at the 3-mm superior sector and 0.341 at the 6-mm superior sector, a relative decline of 64%. For HRA–NIDEK, the decline was from ICC = 0.902 (central) to 0.714 (3-mm superior) to 0.492 (6-mm superior), representing a relative reduction of 45%. For HRA–BMIZAR and HRA–TOPCON, which began with only moderate central agreement (ICC ~ 0.49–0.51), values fell to the 0.30–0.45 range at the 6-mm ring.

**Fig. 1:**
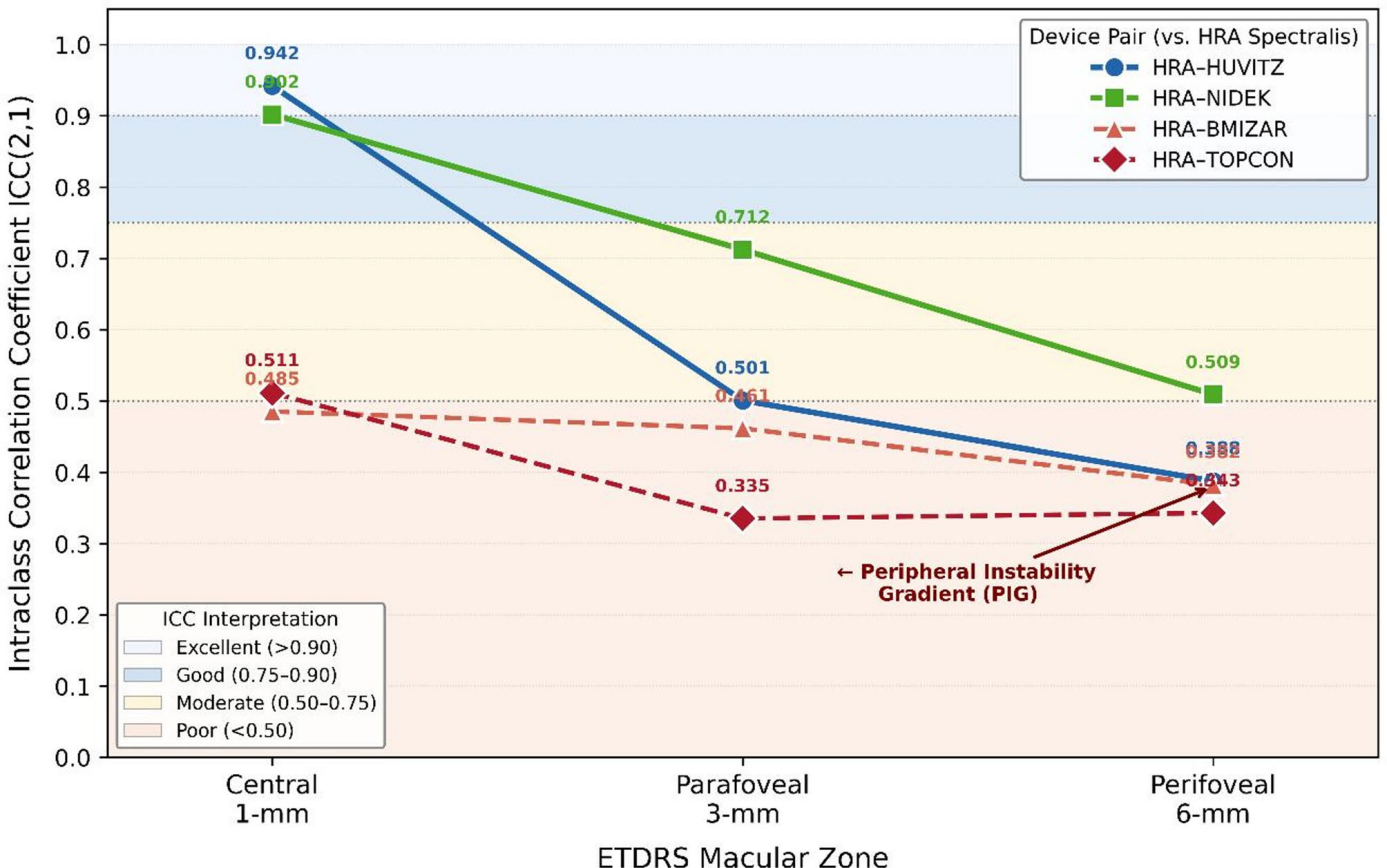
The peripheral instability gradient (PIG): inter-device ICC(2,1) values across ETDRS macular zones. Intraclass correlation coefficients [ICC(2,1)] for inter-device agreement between HRA Spectralis (reference) and each comparator device are plotted against three ETDRS macular zones: central 1-mm subfield, parafoveal 3-mm ring (mean of four sectors), and perifoveal 6-mm ring (mean of four sectors). Coloured background bands indicate ICC interpretation thresholds: excellent (> 0.90, blue), good (0.75–0.90, light blue), moderate (0.50–0.75, yellow), and poor (< 0.50, orange). All device pairs demonstrate progressive deterioration of inter-device agreement from the central subfield outward, confirming the PIG as a robust, device-independent spatial phenomenon. HRA–HUVITZ and HRA–NIDEK begin in the excellent range centrally but decline to the poor-to-moderate range at the 6-mm perifoveal ring, representing ICC declines of 64% and 45%, respectively. HRA–BMIZAR and HRA–TOPCON show only moderate central agreement, declining further to predominantly poor agreement at the 6-mm ring. This stepwise, spatially-structured deterioration is the defining visual representation of the Peripheral Instability Gradient: a predictable, device-independent loss of cross-platform measurement reliability as the ETDRS measurement zone expands from fovea to periphery. ICC: intraclass correlation coefficient; ETDRS: Early Treatment Diabetic Retinopathy Study; HRA: Heidelberg Retina Angiograph Spectralis; PIG: Peripheral Instability Gradient; SD-OCT: spectral-domain OCT; SS-OCTA: swept-source OCT angiography

**Fig. 2:**
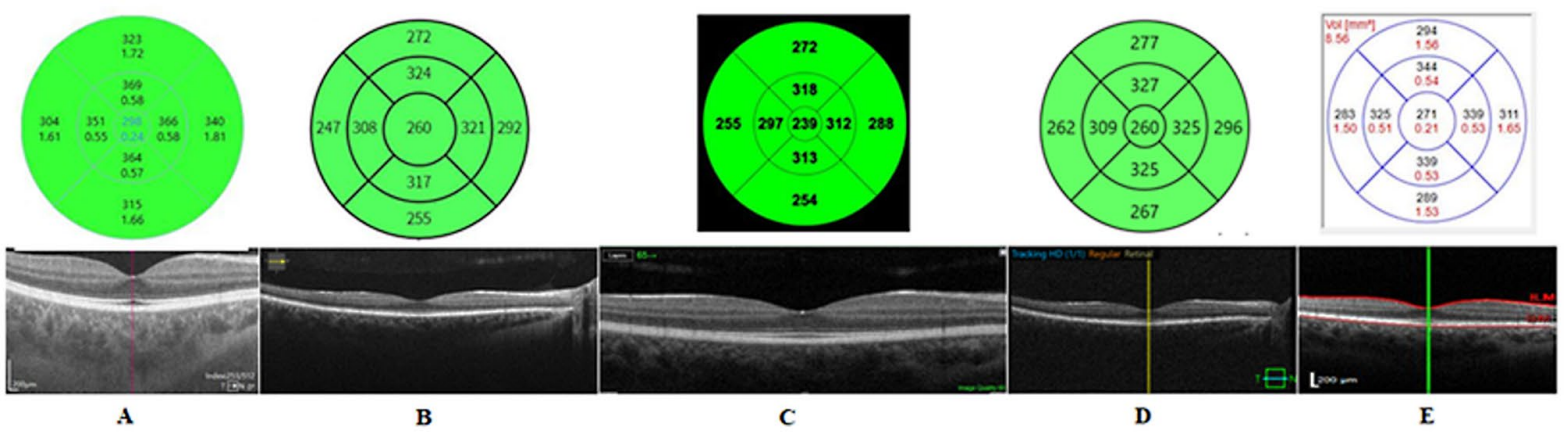
Representative macular ETDRS thickness maps and corresponding cross-sectional OCT B-scans obtained from a single participant across all five OCT platforms. Each panel shows the device-generated output for: (**A**) TowardPi B-Mizar (TowardPi Medical Technology, China), retinal thickness defined from the internal limiting membrane (ILM) to Bruch’s membrane (BM); (**B**) Huvitz OCT-1 F (Huvitz Co., Ltd., South Korea), macular thickness measured between the ILM and the retinal pigment epithelium (RPE); (**C**) Topcon Maestro2 (Topcon Corporation, Japan), macular thickness defined as the distance between the ILM and the outer segment/RPE (OS/RPE) boundary; (**D**) NIDEK RS-1 Glauvas OCT (Nidek Co., Ltd., Japan), full retinal thickness measured from the ILM to the RPE/Bruch’s membrane (RPE/BM) complex; (**E**) Heidelberg Spectralis (Heidelberg Engineering, Germany), total retinal thickness measured between the ILM and BM. The variability in macular thickness values across devices reflects differences in outer retinal boundary definitions and should be carefully considered when comparing quantitative macular thickness measurements across platforms. This figure is intended for qualitative illustration only; quantitative inter-device differences are reported in Table 3. ILM: inner limiting membrane; RPE: retinal pigment epithelium; ETDRS: Early Treatment Diabetic Retinopathy Study

Concordantly, LoA widths expanded progressively with ring diameter. For HUVITZ, LoA widths increased from 7.15% (central) to approximately 8.18% (3-mm superior) and 7.95–21.27% (6-mm ring, by sector). For BMIZAR, the central LoA of 12.6% expanded to 9.58% (3-mm superior) and 5.12–8.92% (6-mm ring); the apparent narrowing in some 6-mm sectors reflects a reduction in absolute µm measurement range rather than improved agreement, as ICC and APE continued to worsen. Median APE increased from its central-subfield minimum (HUVITZ: 2.39%; NIDEK: 3.69%) to values exceeding 10% for HUVITZ and TOPCON at peripheral 6-mm sectors, representing a 4–5-fold increase. Collectively, these findings confirm the PIG as a robust, device-independent phenomenon: the progressive spatial deterioration of cross-device measurement concordance from foveal center to peripheral macular zones [8, 9]. Figure 1 provides a direct visual representation of this stepwise deterioration, plotting ICC(2,1) values for all four device pairs across the three ETDRS zones against ICC interpretation threshold bands, and is recommended as a clinical reference tool for assessing cross-device comparability at a glance. To complement this quantitative characterization, Fig. 2 provides representative macular ETDRS thickness maps and corresponding foveal B-scan cross-sections from the same participant imaged on all five devices, illustrating qualitative cross-platform differences in color-map thickness representation and retinal boundary segmentation placement that are directly concordant with the systematic biases quantified in Table 3.

### All-pairwise cross-validation among five OCT devices

To ensure that inter-device comparability was not dependent on HRA as a single reference device, we performed all 10 direct pairwise comparisons among the five OCT systems at the eye level. The all-pairwise analysis confirmed the same spatial pattern observed in the HRA-anchored analyses: agreement was highest in the central 1-mm subfield and generally deteriorated toward the 3-mm and 6-mm ETDRS sectors. Central agreement was strongest for HUVITZ–NIDEK (ICC = 0.951), HRA–HUVITZ (ICC = 0.936), and HRA–NIDEK (ICC = 0.894), whereas combinations involving BMIZAR or TOPCON showed larger absolute biases. At the 6-mm ring, several pairs showed reduced ICC values and wider Bland–Altman limits, confirming that the Peripheral Instability Gradient was not an artifact of choosing HRA as the reference device. Complete all-pairwise ICC, bias, and limits-of-agreement results are provided in Supplementary Table S1.

### Intra-device repeatability

All five OCT systems demonstrated excellent within-device measurement consistency, with ICC(3,1) values exceeding 0.85 across all regions and devices. Repeated-measures ANOVA of three consecutive measurements revealed no statistically significant differences among R1–R3 and L1–L3 measurements in the vast majority of device–region combinations, confirming high intra-session reproducibility.

#### Central subfield (1-mm)

No significant differences were detected across consecutive measurements for any device in either the right eye (HRA: *p* = 0.693; BMIZAR: *p* = 0.440; HUVITZ: *p* = 0.367; NIDEK: *p* = 0.368; TOPCON: *p* = 0.526) or the left eye (HRA: *p* = 0.628; BMIZAR: *p* = 0.385; HUVITZ: *p* = 0.076; NIDEK: *p* = 0.449; TOPCON: *p* = 0.924).

#### 3-mm ring

In the superior and nasal sectors, no significant intra-device differences were observed for any device in either eye (all *p* > 0.06). Two isolated exceptions were identified: BMIZAR showed a significant difference across consecutive right-eye measurements in the **inferior sector** (*p* = 0.032), and NIDEK demonstrated a significant difference in the right-eye **temporal sector** (*p* = 0.012), with post hoc analysis identifying the R1–R3 pair as the source of the difference (*p* = 0.019) while R2–R3 remained non-significant (*p* = 0.999). No corresponding differences were found in the left eye for either device. One additional observation is the markedly elevated SD for TOPCON Superior 3-mm R3 (47.85 μm) compared with R1 (~ 16 μm) and R2 (~ 16 μm); this reflects an isolated outlying third-repetition measurement in one participant verified in the raw dataset and retained as recorded—the overall ANOVA p-value for this combination (*p* = 0.201) remained non-significant, confirming the absence of systematic intra-device instability for TOPCON in this sector (Table 5, footnote†).

#### 6-mm ring

No statistically significant differences across consecutive measurements were detected for any device in any sector, for either the right or left eye (all *p* > 0.09), indicating that intra-device stability is fully maintained even at the most peripheral macular zone evaluated (Tables 5 and 6).

These findings underscore that device-switching, rather than within-device measurement noise, is the primary contributor to macular thickness discrepancy in OCT-based longitudinal monitoring. The excellent within-device repeatability of all five systems provides reassurance that, when patients are followed on the same device with the same protocol, apparent thickness changes can be reliably attributed to true anatomical change rather than instrument-related variability [4, 5, 10].

**Table 4:** Comparison of inner macular thickness (6-mm ETDRS) measurements across five devices

(*n* = 38)	I	II	III	IV	V	*p* Value ᵃ	Pairwise Comparisons			
	HRA	BMIZAR	HUVITZ	NIDEK	TOPCON					
	Mean (SD.)	Mean (SD.)	Mean (SD.)	Mean (SD.)	Mean (SD.)		I vs. II	I vs. III	I vs. IV	I vs. V
**Inner Macular Thickness (6-mm) (µm)**										
** Superior**										
Mean of Right and Left Eyes (µm)	306.00 (15.07)	331.89 (14.70)	278.58 (13.81)	289.07 (13.33)	278.45 (14.27)	< 0.001	< 0.001	< 0.001	< 0.001	< 0.001
Right Eye Mean (Rep 1–3) (µm)	305.66 (15.02)	331.75 (14.19)	277.98 (13.94)	289.51 (13.47)	277.75 (14.36)	< 0.001	< 0.001	< 0.001	< 0.001	< 0.001
Left Eye Mean (Rep 1–3) (µm)	306.35 (15.39)	332.02 (15.71)	279.18 (14.89)	288.63 (13.46)	279.14 (14.61)	< 0.001	< 0.001	< 0.001	< 0.001	< 0.001
** P value (Right vs. Left) **ᵗ	0.312	0.796	0.376	0.167	0.094					
** Nasal**										
Mean of Right and Left Eyes (µm)	320.20 (15.91)	348.11 (16.96)	295.99 (17.38)	307.95 (15.45)	291.83 (15.16)	< 0.001	< 0.001	< 0.001	< 0.001	< 0.001
Right Eye Mean (Rep 1–3) (µm)	319.17 (15.62)	346.18 (16.28)	296.66 (14.63)	307.18 (14.80)	291.76 (14.99)	< 0.001	< 0.001	< 0.001	< 0.001	< 0.001
Left Eye Mean (Rep 1–3) (µm)	321.23 (16.60)	350.04 (18.51)	295.32 (23.80)	308.71 (16.28)	291.89 (16.80)	< 0.001	< 0.001	< 0.001	< 0.001	< 0.001
** P value (Right vs. Left) **ᵗ	**0.027**	0.019	0.680	**0.015**	0.938					
** Inferior**										
Mean of Right and Left Eyes (µm)	295.46 (14.32)	324.04 (16.07)	266.75 (13.45)	279.65 (14.68)	268.38 (13.90)	< 0.001	< 0.001	< 0.001	< 0.001	< 0.001
Right Eye Mean (Rep 1–3) (µm)	295.13 (14.08)	322.48 (14.69)	265.74 (14.55)	278.68 (14.93)	268.13 (13.75)	< 0.001	< 0.001	< 0.001	< 0.001	< 0.001
Left Eye Mean (Rep 1–3) (µm)	295.78 (15.10)	325.60 (19.89)	267.76 (14.24)	280.62 (15.90)	268.63 (15.26)	< 0.001	< 0.001	< 0.001	< 0.001	< 0.001
** P value (Right vs. Left) **ᵗ	0.489	0.241	0.227	0.222	0.713					
** Temporal**										
Mean of Right and Left Eyes (µm)	290.14 (15.04)	312.23 (16.07)	259.05 (14.05)	270.27 (12.88)	262.84 (14.47)	< 0.001	< 0.001	< 0.001	< 0.001	< 0.001
Right Eye Mean (Rep 1–3) (µm)	290.28 (14.93)	312.18 (14.55)	259.18 (14.26)	270.01 (12.67)	262.14 (15.10)	< 0.001	< 0.001	< 0.001	< 0.001	< 0.001
Left Eye Mean (Rep 1–3) (µm)	290.01 (15.58)	312.28 (18.61)	258.92 (16.61)	270.54 (13.24)	263.54 (15.81)	< 0.001	< 0.001	< 0.001	< 0.001	< 0.001
** P value (Right vs. Left) **ᵗ	0.756	0.947	0.888	0.252	0.450					

ᵃ Repeated measures analysis of variance (ANOVA) was performed to evaluate the differences in measurement results between five devices in the same patients, Adjustment for multiple comparisons: Bonferroni, ᵗ Paired Samples t-test (Bootsrap), SD. Standard Deviation, R1–R3 and L1–L3 represent three repeated measurements obtained from the right and left eyes, respectively

**Table 5:** Intra-device repeatability of central (1-mm) and inner (3-mm) ETDRS macular thickness measurements based on three consecutive measurements

	HRA	BMIZAR	HUVITZ	NIDEK	TOPCON
	Mean (SD.)	Mean (SD.)	Mean (SD.)	Mean (SD.)	Mean (SD.)
**Central Macular Thickness (1-mm) (µm)**					
R1	258.29 (23.07)	288.26 (23.66)	252.47 (23.69)	251.79 (28.65)	227.21 (22.89)
R2	258.42 (23.28)	290.45 (27.34)	250.18 (23.67)	249.26 (22.58)	227.74 (22.50)
R3	258.21 (23.30)	288.34 (23.26)	251.95 (21.70)	249.45 (22.56)	227.18 (23.76)
** P value (R1 vs. R2 vs. R3)**	0.693	0.44	0.367	0.368	0.526
L1	258.92 (22.53)	291.16 (28.81)	252.95 (21.57)	249.03 (22.02)	228.37 (22.57)
L2	259.03 (22.48)	294.00 (31.73)	252.82 (21.99)	249.26 (22.02)	228.21 (22.83)
L3	258.84 (22.57)	292.16 (29.90)	252.00 (22.01)	249.16 (21.81)	228.37 (22.37)
** P value (L1 vs. L2 vs. L3)**	0.628	0.385	0.076	0.449	0.924
**Inner Macular Thickness (3-mm) (µm)**					
** Superior**					
R1	344.21 (24.21)	370.71 (17.21)	323.61 (16.34)	334.37 (16.44)	315.87 (16.77)
R2	347.16 (17.16)	370.39 (17.34)	322.63 (16.94)	334.42 (16.85)	316.58 (16.51)
R3	346.71 (17.45)	370.74 (17.21)	319.97 (26.75)	334.39 (16.34)	305.66 (47.85)
** P value (R1 vs. R2 vs. R3)**	0.304	0.53	0.402	0.961	0.201
L1	346.92 (16.98)	371.37 (18.73)	322.26 (16.29)	333.58 (16.34)	314.08 (21.46)
L2	346.34 (16.99)	370.76 (19.03)	322.32 (16.48)	333.74 (16.49)	317.21 (16.55)
L3	346.00 (16.80)	368.21 (26.89)	322.37 (16.43)	333.39 (16.34)	316.13 (16.21)
** P value (L1 vs. L2 vs. L3)**	0.066	0.281	0.964	0.146	0.277
** Nasal**					
R1	343.47 (18.74)	370.50 (17.91)	318.89 (18.56)	331.16 (18.34)	311.82 (18.66)
R2	344.24 (19.09)	370.74 (18.67)	320.11 (18.75)	331.03 (18.43)	312.18 (18.22)
R3	343.61 (19.10)	370.63 (18.56)	321.92 (17.97)	331.05 (18.48)	311.87 (18.25)
** P value (R1 vs. R2 vs. R3)**	0.317	0.853	0.455	0.545	0.699
L1	344.42 (19.39)	372.68 (22.86)	323.32 (18.47)	331.84 (18.52)	313.00 (18.71)
L2	343.95 (19.93)	370.82 (23.05)	323.05 (18.37)	331.95 (18.70)	313.53 (18.55)
L3	343.89 (19.38)	372.74 (22.93)	323.47 (18.32)	331.76 (18.29)	312.89 (19.06)
** P value (L1 vs. L2 vs. L3)**	0.414	0.316	0.759	0.446	0.509
** Inferior**					
R1	342.87 (16.86)	368.71 (16.75)	318.68 (15.16)	330.68 (15.80)	312.89 (16.26)
R2	343.08 (17.20)	368.29 (16.37)	317.95 (15.73)	330.34 (15.69)	312.24 (17.98)
R3	343.05 (16.66)	367.45 (17.02)	318.00 (15.93)	327.87 (20.25)	312.79 (16.95)
** P value (R1 vs. R2 vs. R3)**	0.778	0.032	0.595	0.319	0.686
L1	344.21 (17.81)	370.68 (23.92)	319.87 (16.17)	330.92 (17.03)	313.58 (16.39)
L2	344.47 (17.67)	370.95 (23.45)	320.53 (16.19)	331.03 (17.09)	312.84 (17.58)
L3	344.61 (17.75)	368.71 (17.77)	319.97 (17.04)	330.89 (16.94)	313.42 (16.74)
** P value (L1 vs. L2 vs. L3)**	0.335	0.387	0.45	0.792	0.503
** Temporal**					
R1	331.42 (17.40)	357.18 (18.39)	309.87 (16.81)	317.68 (16.21) ^R3^	301.08 (16.36)
R2	330.97 (17.44)	356.21 (17.02)	309.55 (15.57)	317.34 (16.19)	301.00 (16.42)
R3	331.26 (17.42)	356.24 (17.18)	308.87 (15.13)	317.21 (16.14)	300.32 (16.88)
** P value (R1 vs. R2 vs. R3)**	0.661	0.171	0.518	0.012	0.202
L1	330.76 (17.87)	358.37 (23.60)	310.21 (16.27)	316.89 (17.16)	301.24 (16.73)
L2	331.00 (17.62)	358.37 (22.80)	310.21 (16.32)	316.95 (17.24)	300.58 (16.54)
L3	330.87 (17.68)	358.79 (24.76)	309.39 (16.37)	316.71 (17.20)	301.16 (16.48)
** P value (L1 vs. L2 vs. L3)**	0.707	0.527	0.195	0.197	0.498

Repeated measures analysis of variance (ANOVA) was performed to evaluate differences across the three consecutive measurements, Adjustment for multiple comparisons: Bonferroni, SD. Standard Deviation, Superscript R3 denotes that the statistically significant difference among consecutive measurements was attributable to the third repetition (R3), as identified by post hoc Bonferroni-corrected pairwise comparisons

R1–R3 and L1–L3 represent three repeated measurements obtained from the right and left eyes, respectively

†SD for TOPCON Superior 3-mm R3 (47.85 μm) is markedly larger than R1/R2 (~ 16 μm), reflecting an outlying measurement in one participant’s third repetition. This data point was verified in the raw dataset and is retained as recorded; the overall ANOVA p-value for this row (0.201) remains non-significant, indicating no systematic intra-device instability for TOPCON in this sector

**Table 6:** Intra-device repeatability of inner macular thickness (6-mm ETDRS) measurements based on three consecutive scans

	HRA	BMIZAR	HUVITZ	NIDEK	TOPCON
	Mean (SD.)	Mean (SD.)	Mean (SD.)	Mean (SD.)	Mean (SD.)
Inner Macular Thickness (6-mm) (µm)					
** Superior**					
R1	305.87 (15.03)	332.08 (14.39)	278.79 (14.78)	289.53 (13.30)	277.39 (14.81)
R2	305.58 (15.03)	331.74 (14.10)	277.95 (13.87)	289.45 (13.49)	277.82 (13.99)
R3	305.53 (15.20)	331.45 (14.23)	277.21 (14.60)	289.55 (13.72)	278.05 (14.65)
** P value (R1 vs. R2 vs. R3)**	0.622	0.174	0.317	0.912	0.467
L1	306.55 (15.40)	331.82 (15.31)	280.00 (20.62)	288.76 (13.36)	279.39 (14.68)
L2	306.08 (15.52)	332.00 (15.73)	279.45 (15.41)	288.79 (13.47)	278.87 (14.50)
L3	306.42 (15.34)	332.24 (16.31)	278.08 (15.17)	288.34 (13.63)	279.16 (15.10)
** P value (L1 vs. L2 vs. L3)**	0.22	0.599	0.648	0.126	0.669
** Nasal**					
R1	319.68 (15.21)	345.58 (17.55)	297.39 (14.30)	307.26 (14.77)	291.21 (15.45)
R2	318.71 (16.15)	346.61 (16.36)	297.53 (15.14)	307.21 (14.78)	292.00 (15.66)
R3	319.11 (16.18)	346.34 (16.32)	295.05 (19.61)	307.08 (14.88)	292.08 (15.52)
** P value (R1 vs. R2 vs. R3)**	0.378	0.512	0.417	0.288	0.595
L1	321.42 (16.32)	349.68 (18.09)	292.76 (48.79)	308.66 (16.36)	291.00 (17.91)
L2	320.76 (17.07)	350.00 (18.73)	295.89 (21.02)	308.82 (16.48)	291.87 (18.21)
L3	321.50 (16.77)	350.42 (18.83)	297.32 (19.13)	308.66 (16.03)	292.82 (18.08)
** P value (L1 vs. L2 vs. L3)**	0.337	0.093	0.611	0.478	0.539
** Inferior**					
R1	295.03 (14.16)	322.55 (14.94)	263.84 (14.19)	277.82 (13.25)	266.55 (16.91)
R2	295.29 (14.11)	322.18 (14.60)	266.45 (16.61)	280.58 (22.13)	268.53 (13.19)
R3	295.08 (14.18)	322.71 (14.74)	266.92 (16.21)	277.66 (13.19)	269.32 (13.73)
** P value (R1 vs. R2 vs. R3)**	0.786	0.395	0.143	0.28	0.174
L1	295.58 (15.41)	325.26 (20.05)	269.97 (17.46)	282.37 (23.26)	269.58 (17.59)
L2	296.08 (15.07)	325.29 (19.27)	266.79 (15.40)	279.79 (14.13)	268.42 (15.03)
L3	295.68 (14.99)	326.24 (20.60)	266.53 (15.20)	279.71 (14.10)	267.89 (14.53)
** P value (L1 vs. L2 vs. L3)**	0.356	0.113	0.2	0.335	0.297
** Temporal**					
R1	289.39 (14.75)	313.18 (16.31)	256.87 (16.83)	270.11 (12.75)	262.11 (16.71)
R2	290.97 (15.32)	311.82 (15.20)	257.42 (18.16)	269.87 (12.57)	262.68 (15.61)
R3	290.47 (15.46)	311.53 (13.88)	263.26 (21.26)	270.05 (12.75)	261.63 (14.96)
** P value (R1 vs. R2 vs. R3)**	0.134	0.322	0.147	0.411	0.654
L1	289.71 (15.91)	313.11 (17.57)	258.79 (16.91)	270.53 (13.30)	265.37 (18.47)
L2	290.45 (16.34)	312.71 (16.70)	259.63 (18.37)	270.66 (13.38)	263.55 (16.81)
L3	289.87 (14.94)	311.03 (23.60)	258.34 (16.55)	270.42 (13.07)	261.68 (15.01)
** P value (L1 vs. L2 vs. L3)**	0.444	0.359	0.597	0.373	0.095

Repeated measures analysis of variance (ANOVA) was performed to evaluate differences across the three consecutive measurements, Adjustment for multiple comparisons: Bonferroni, SD. Standard Deviation

R1–R3 and L1–L3 represent three repeated measurements obtained from the right and left eyes, respectively

## Discussion

This study systematically evaluated intra-device repeatability and inter-device agreement of macular thickness measurements across five contemporary OCT platforms in healthy adults. The cardinal finding is the Peripheral Instability Gradient (PIG), a robust, device-independent, spatially progressive deterioration in cross-device measurement concordance from the foveal center outward, observed against a background of uniformly high within-device repeatability (ICC(3,1) > 0.85 across all regions and devices. However, inter-device agreement was strongly location-dependent: ICC(2,1) values declined by a relative 40–67% from the central 1-mm subfield to the 6-mm ring, LoA widths expanded by a relative 30–50%, and median APE increased up to fivefold in peripheral sectors. These findings demonstrate that while macular thickness measurements are internally consistent within individual OCT systems, they are substantially non-interchangeable across platforms—particularly beyond the central macular subfield. Importantly, the all-pairwise supplementary cross-validation analysis confirmed that this conclusion was not dependent on using HRA as the reference device. Across all 10 device pairs, the same central-to-peripheral deterioration in agreement was observed, with the highest central agreement occurring between HUVITZ–NIDEK, HRA–HUVITZ, and HRA–NIDEK, and weaker agreement in several pairings involving BMIZAR or TOPCON. Thus, HRA served only as an interpretive anchor for directional bias in the main tables, whereas the broader conclusion of limited inter-device interchangeability is supported by reference-independent pairwise comparisons.

Before discussing these findings in detail, it is important to contextualize the study population. By enrolling only young healthy adults with strict image quality criteria, this study establishes PIG magnitude estimates under optimal conditions. The agreement values reported here—including the 40–67% ICC decline and 30–50% LoA widening from central to peripheral ETDRS zones—therefore represent the most favorable scenario and should be considered conservative lower bounds of inter-device disagreement. In eyes affected by macular disease, where segmentation algorithm robustness is challenged by structural disruption, the PIG is expected to be more pronounced, and the central-to-peripheral ICC gradient steeper. The clinical framework introduced here provides the methodological foundation for future validation studies in pathological populations.

All five OCT systems—including BMIZAR, evaluated here in a multi-device context for the first time—demonstrated high within-device measurement consistency. Sequential measurements acquired during a single session showed no statistically significant differences across the vast majority of device–sector combinations (all *p* > 0.09 at the 6-mm ring; isolated exceptions at the 3-mm ring for BMIZAR inferior [*p* = 0.032] and NIDEK temporal [*p* = 0.012]). ICC(3,1) values consistently exceeded 0.85 across all regions.

This finding provides critical clinical reassurance: when a patient is imaged on the same OCT device using the same standardized protocol, apparent changes in macular thickness reliably reflect true anatomical change rather than instrument-related variability. Prior repeatability studies corroborate this stability—ETDRS-based macular thickness repeatability remains high within a given instrument, although test–retest variability may modestly increase toward more peripheral rings [4, 5, 14]. The inclusion of BMIZAR in this assessment is particularly informative: despite its swept-source OCTA architecture differing fundamentally from the spectral-domain devices in the comparison, within-session repeatability was preserved, supporting its use for serial monitoring when device consistency is maintained.

The two isolated exceptions to excellent repeatability—BMIZAR’s inferior 3-mm sector and NIDEK’s temporal 3-mm sector—warrant brief comment. Post hoc analysis for NIDEK identified the R1–R3 pair as the source of the temporal difference (*p* = 0.019), while R2–R3 was non-significant (*p* = 0.999), suggesting a possible first-measurement familiarization effect rather than true instrument instability. Neither exception was replicated in the contralateral eye, and no significant differences were observed at the 6-mm ring for either device, further supporting their classification as isolated rather than systematic phenomena.

### Inter-device variability and the peripheral instability gradient

Contrary to within-device stability, substantial and systematic inter-device differences were observed that varied predictably by macular location—the defining characteristic of the PIG. This gradient was consistent across all device pairs and represents the study’s most clinically significant contribution.

At the foveal center (central 1-mm subfield), inter-device agreement was highest across the study, with ICC values reaching the excellent range for HRA–HUVITZ (0.942) and HRA–NIDEK (0.902). These values indicate that, in the best-case scenario, approximately 80–90% of the observed variance between devices is attributable to true biological differences among subjects rather than device-related noise—the hallmark of acceptable cross-device comparability. HRA–BMIZAR (ICC = 0.485) and HRA–TOPCON (ICC = 0.511) fell in the moderate range, reflecting larger systematic differences that limit direct interchangeability even centrally. In the context of the broader OCT literature, these central ICC values are consistent with prior cross-platform studies: Nam et al. (2024) and Hanumunthadu et al. (2021) similarly reported higher central agreement between SD-OCT and SS-OCT platforms, while inter-device agreement remained imperfect even in this most favorable region [7, 9].

The PIG became clearly manifest at 3-mm parafoveal ring level: ICC values declined substantially for all device pairs relative to the central subfield. Even the best-performing pair (HRA–NIDEK) fell from ICC = 0.902 to the 0.67–0.76 range across sectors—a shift from excellent to moderate-to-good reliability. HRA–BMIZAR and HRA–HUVITZ declined to the low-to-moderate range (0.43–0.54), and HRA–TOPCON fell predominantly to poor-to-moderate agreement (0.20–0.39). This deterioration occurred despite identical acquisition conditions across ETDRS rings, confirming that the decline in agreement is spatially driven; a function of measurement location rather than extrinsic confounders.

The 6-mm perifoveal ring represented the nadir of inter-device agreement, demonstrating the full magnitude of the PIG. HRA–NIDEK, the strongest-performing pair, fell to low-to-moderate agreement (ICC 0.40–0.67), while agreement for the remaining pairs was predominantly poor (< 0.40). LoA widths widened by 30–50% relative to the central subfield (e.g., HUVITZ LoA rising from 7.15% to 21.27% at the 6-mm nasal sector), and median APE values reached their maximum (HUVITZ temporal: 11.46%; TOPCON superior: 8.87%). These findings align with and extend the observations of Hou et al. (2023) and Chalam et al. (2025), who reported greater inter-device discrepancy in outer ETDRS sectors relative to the central subfield in both healthy and glaucomatous eyes [8, 11].

The spatial pattern of the PIG is consistent with well-established properties of retinal OCT imaging: inter-device agreement is maximized where the retinal architecture is most stereotyped (the fovea), lamination is most distinct, and segmentation algorithms are most reliably calibrated. As the measurement region expands peripherally, the cumulative effects of device-specific technical differences compound progressively, as illustrated in Fig. 1 [7, 8, 10].

### Technical determinants of the peripheral instability gradient

Several interconnected technical factors likely underlie the PIG, each becoming more impactful as the measurement region expands toward the retinal periphery:

Segmentation algorithm differences. Automated segmentation is the principal source of inter-device disagreement in OCT macular thickness measurement. Algorithms are predominantly optimized for the foveal region, where the ILM and RPE boundaries are clearly delineated, and lamination is most distinct. In peripheral macular zones, decreasing layer contrast, increasing retinal curvature, and variable RPE pigmentation impair boundary detection, introducing device-specific segmentation errors that are not present or are substantially smaller centrally. Critically, different manufacturers define the outer retinal boundary differently (e.g., inner RPE surface vs. Bruch’s membrane vs. photoreceptor outer segment tip), and these definitional differences produce larger absolute discrepancies as retinal thickness and curvature increase peripherally [2, 3]. Bland–Altman analysis confirms that inter-device disagreement in this study is predominantly systematic rather than random, consistent with reproducible differences in measurement models rather than stochastic noise [13].

Eye-tracking and fixation stability. HRA Spectralis employs active TruTrack eye-tracking during acquisition, enabling precise scan registration and minimizing fixation-decentration artifacts. Devices without equivalent active tracking systems are susceptible to fixation variability between acquisitions, which introduces greater measurement error in curved peripheral retinal regions compared with the flatter foveal center [8]. This technical asymmetry is likely a meaningful contributor to the PIG, as fixation decentration of even 0.5° can shift ETDRS ring boundaries by ~ 130 μm at the 3-mm ring and ~ 260 μm at the 6-mm ring, introducing sector-reassignment artifacts that are not detectable from thickness values alone.

Scan density and averaging protocols. B-scan density, A-scan acquisition frequency, and frame averaging differ across platforms and directly affect measurement precision in regions with greater anatomical heterogeneity. At the 6-mm ring, individual B-scans sample more anatomically variable territory—including larger retinal vessels, more pronounced drusen-like deposits in healthy retinas, and increasing topographic complexity—than at the foveal center. Device-specific differences in how this heterogeneity is averaged or suppressed translate into differential measurement outputs that are amplified relative to central measurements [10].

Retinal curvature and vessel shadowing. Toward the parafoveal and perifoveal regions, the retinal surface curves more steeply, and retinal vessels cast increasingly prominent shadows. These optical factors impair automated boundary detection in a device-specific manner, contributing to directional biases that increase with eccentricity [8, 15]. The progressive widening of LoA values from central to 6-mm rings directly reflects these compounding influences.

### Device-specific technical profiles and their relationship to observed bias direction

The directional biases observed across devices are not arbitrary. They reflect predictable consequences of each platform’s measurement architecture and outer retinal boundary definition conventions:

BMIZAR (TowardPi, 400 kHz SS-OCTA) systematically overestimates relative to HRA and employs swept-source technology with a longer central wavelength (~ 1050 nm) than the spectral-domain devices in this study (~ 840 nm). The longer wavelength enables deeper penetration into choroidal tissue, and the high acquisition speed (400 kHz A-scans/second) permits dense volumetric sampling using repeated B-scan averaging for motion-contrast analysis. However, the outer retinal segmentation boundary in SS-OCTA platforms is particularly susceptible to ambiguity at the level of the ellipsoid zone and outer segment–RPE interface, where the signal-to-noise characteristics differ fundamentally from SD-OCT. If BMIZAR’s proprietary segmentation places the outer boundary at a deeper layer relative to HRA’s outer boundary definition, this would produce systematically greater measured thickness values across all zones. The proportional bias observed for BMIZAR (*p* = 0.010 centrally) further indicates that this overestimation scales with absolute retinal thickness, consistent with a boundary-definition offset that becomes larger in absolute terms where the retina is thicker. Additionally, the widefield acquisition geometry of BMIZAR means that peripheral zones are imaged at steeper angles of incidence, which can artificially inflate apparent layer thickness due to the non-perpendicular optical path [2, 3].

TOPCON Maestro 2 systematically underestimates relative to HRA, and its fully automated acquisition pipeline integrates autofocus, automated alignment, and single-capture volumetric scanning designed to optimize patient throughput. While this approach ensures consistent acquisition, its proprietary segmentation algorithm may define the outer retinal boundary at a shallower depth, resulting in systematically lower thickness measurements compared to HRA’s segmentation convention. Significant proportional bias (*p* < 0.001) indicates that TOPCON’s underestimation scales with thickness, consistent with a systematic definitional difference rather than random acquisition variability. The lower B-scan density achievable in a single automated capture compared to HRA’s TruTrack-assisted multi-frame averaging may also reduce the accuracy of automated boundary detection at the RPE level, particularly in peripheral zones where retinal curvature and vessel shadowing are most pronounced [7, 9].

HUVITZ and NIDEK have closer alignments with HRA, and both platforms operate as spectral-domain OCT systems near the same wavelength range as HRA (~ 840 nm), and their segmentation algorithms appear to define the ILM-to-RPE boundary in a manner more closely approximating HRA’s convention, resulting in smaller mean biases (HUVITZ: +2.60%; NIDEK: +3.64% centrally). The slight positive bias (HRA exceeds comparator) for both devices suggests a minor tendency toward RPE boundary placement slightly interior to HRA’s definition, but of a magnitude unlikely to affect most routine clinical decisions. The observation that NIDEK maintains the most consistent agreement profile across peripheral zones may reflect a higher effective scan density or a segmentation approach that is more robust to the peripheral reduction in retinal layer contrast compared with HUVITZ.

### Systematic bias and its clinical implications

Bland–Altman analysis revealed that inter-device disagreement is predominantly systematic rather than random, with directional biases reflecting fundamental differences in device measurement models. The directionality and magnitude of bias were consistent across ETDRS rings for each device, confirming their device-specific rather than sector-specific nature.

**BMIZAR** systematically overestimated central macular thickness relative to HRA by approximately 10.98% (equivalent to ~ 28 μm at a mean thickness of 258 μm), with significant proportional bias indicating that the degree of overestimation increases with absolute retinal thickness (*p* = 0.010). Across the 3-mm and 6-mm rings, BMIZAR’s relative overestimation bias was maintained (HRA vs. BMIZAR range: −6.49% to − 8.78%), consistent with a global tendency to measure a thicker retina than HRA, possibly related to differences in the SS-OCTA acquisition pipeline and outer boundary definition.

**TOPCON** exhibited the systematic underestimation bias relative to HRA (HRA vs. TOPCON: +13.65% centrally, meaning TOPCON underestimates by ~ 13.65%; ~35 μm), also with significant proportional bias (*p* < 0.001). Central thickness values exceeding 300 μm are commonly used as retreatment thresholds for anti-VEGF therapy in DME; a relative + 14% underestimation by TOPCON relative to HRA could lead to under-treatment by falsely suggesting edema resolution. This finding is consistent with prior reports documenting TOPCON-specific measurement values [9, 11].

**HUVITZ and NIDEK** demonstrated substantially more favorable central agreement profiles (HUVITZ: +2.60%, LoA 7.15%; NIDEK: +3.64%, LoA 9.39%), with biases of a magnitude less likely to affect routine clinical decisions. Notably, HUVITZ showed a significant proportional bias (*p* = 0.042) despite its small mean bias, indicating that agreement is not uniform across the thickness range—a finding that warrants consideration when monitoring retinas at the extremes of physiological thickness. NIDEK maintained the most favorable and consistent agreement profile across both the 3-mm and 6-mm rings, demonstrating the narrowest LoA widths and lowest APE values at peripheral locations among the comparator devices.

An important methodological consideration is the dependency of all reported bias estimates on HRA as the reference device. If HRA itself carries a systematic offset relative to an absolute anatomical standard, all reported biases would shift in magnitude accordingly. However, the relative inter-device structure of biases is a property of the devices themselves, not of the reference choice. Because all comparators were benchmarked against the same HRA reference simultaneously, a fixed HRA offset would affect every comparator equally, leaving the relative ordering invariant. The systematic, reproducible, and proportional nature of the observed biases further argues against these being reference-noise artifacts; they reflect stable, device architecture-driven measurement model differences that would manifest regardless of which device served as anchor. Clinicians should therefore interpret the reported bias estimates as relative calibration factors between platforms rather than absolute deviations from biological ground truth.

At the 6-mm ring, inter-device differences were most pronounced: HUVITZ temporal bias reached + 12.09% with a LoA width of 13.44%, which means a true HRA-measured thickness of ~ 290 μm could plausibly yield 255–325 μm on HUVITZ, a 70 μm range. In conditions such as toxicity screening for chloroquine/hydroxychloroquine retinopathy or early perifoveal involvement in retinal dystrophies, where treatment or surveillance decisions hinge on detecting subtle perifoveal thinning, device-related variation of this magnitude is clinically unacceptable without explicit calibration [16]. These findings reinforce the importance of recognizing that applying cutoffs or normative thresholds derived from one platform to measurements obtained on another is inherently problematic [15].

The present findings align with and extend established evidence on cross-platform OCT variability. Macular thickness measurements were compared between time-domain OCT (Stratus OCT) and spectral-domain OCT (3D OCT, Topcon) in 35 healthy subjects [17], demonstrating that while both platforms showed acceptable repeatability, measurements could not be used interchangeably [18]. Specifically, 3D OCT produced significantly greater foveal thickness values (mean difference 20.8 μm; 95% LoA span 33.9 μm), with SD-OCT exhibiting superior repeatability across all sectors (all *P* < 0.014)0.1 These inter-device discrepancies—rooted in differing outer retinal boundary definitions—directly corroborate the spatial variability patterns quantified in our five-platform framework.

Multiple commercially available SD-OCT platforms and emphasized that retinal thickness measurements are fundamentally dependent on how each system segments the outer retinal border, with discrepancies reaching up to 70–80 μm between instruments [17]. They further noted that the Spectralis HRA + OCT achieves superior signal-to-noise ratio through real-time eye-tracking and B-scan averaging—a technical advantage consistent with its relatively stable cross-sector performance observed in the present study.2 Collectively, their review confirms that no two OCT platforms can be considered interchangeable for retinal thickness monitoring, a conclusion our five-device analysis spatially stratifies through explicit Peripheral Instability Gradient (PIG) quantification.

Collectively, this study is the first to simultaneously evaluate five contemporary OCT platforms including a 400 kHz swept-source OCTA system (BMIZAR), to explicitly define and quantify the PIG as a named measurable construct, and to translate these findings into zone-specific clinical guidance.

### Device-specific guidance for clinical practice

Findings of the study provide an evidence base for device selection and longitudinal monitoring strategies:

**HUVITZ HOCT-1/1F** demonstrated the closest central agreement with HRA (ICC = 0.942; APE 2.39%), the best performance of any comparator at the 1-mm subfield. This profile supports its use in general ophthalmic practice for central macular thickness monitoring in conditions such as DME, where central subfield measurements drive treatment decisions. However, its agreement deteriorates substantially at the 6-mm ring, and clinicians should exercise caution when using HUVITZ-derived peripheral measurements for longitudinal tracking without device-specific baselines.

**NIDEK RS-1** demonstrated the most consistent cross-region performance, with moderate-to-good ICC across 3-mm sectors (0.67–0.76) and low-to-moderate ICC at the 6-mm ring (0.40–0.67), superior to all other comparators at peripheral zones. Median APE remained the lowest of any comparator across all ETDRS regions (3.69–6.10%). This profile recommends NIDEK for practices requiring reliable parafoveal and perifoveal monitoring, such as toxicity screening or neurodegenerative disease follow-up.

**BMIZAR (TowardPi**,** 400 kHz SS-OCTA)** showed larger systematic biases (overestimating HRA by ~ 10.98% centrally; HRA vs. BMIZAR: −10.98%) and lower ICC values than HUVITZ or NIDEK, reflecting its fundamentally different measurement model and wider-field acquisition approach. However, its unique capabilities, including widefield OCTA, ultra-high acquisition speed, and deep tissue penetration, may justify its use in specialized settings requiring multimodal structural and angiographic data. When BMIZAR-derived thickness values are used for clinical decisions, device-specific baselines relative to HRA (or equivalent calibration factors) are essential.

**TOPCON Maestro 2** exhibited the systematic underestimation bias (HRA vs. TOPCON: +13.65% centrally; meaning TOPCON underestimates HRA by ~ 13.65%; +10–12% peripherally) with significant proportional bias, indicating that its measurement model differs most substantially from HRA among the evaluated devices. Expect ~ 14% lower central readings on TOPCON compared with HRA; apply bias-correction factors accordingly.

To provide a directly actionable clinical reference, Table 7 summarizes the mean bias, LoA width, and ICC-based comparability classification for each device pair across the three ETDRS zones, with explicit guidance on which zones allow cautious cross-device comparison and which require mandatory device-specific baseline recalibration.

**Table 7:** Clinical summary of inter-device bias and ETDRS zone-specific comparability guidance

Device Pair	Central 1-mm	3-mm Ring	6-mm Ring
	Bias / LoA / ICC	Bias / LoA / ICC	Bias / LoA / ICC
HRA vs. HUVITZ	+ 2.60%/7.15%/0.942. *	+ 6.9–7.7%/5.7–8.2%/0.46–0.54. #	+ 8.4–12.1%/7.9–21.3%/0.28–0.42. x
HRA vs. NIDEK	+ 3.64%/9.39%/0.902. *	+ 3.7–4.4%/7.6–9.6%/0.67–0.76. #	+ 4.0–7.4%/10.8–14.3%/0.40–0.66. #
HRA vs. BMIZAR	−10.98%/12.6%/0.485. #	−6.5 to − 7.4%/8.4–10.4%/0.43–0.47. x	−7.0 to − 8.8%/5.1–8.9%/0.32–0.45. x
HRA vs. TOPCON	+ 13.65%/9.73%/0.511. #	+ 9.9–10.3%/7.3–21.0%/0.27–0.39. x	+ 9.8–10.5%/6.3–11.5%/0.34–0.35. x

*: **Cautious cross-device comparison acceptable** (ICC ≥ 0.90; Bias < 5%; LoA < 10%) with explicit documentation of device change and awareness of known bias direction

#: **Cross-device comparison requires caution** (ICC 0.50–0.90; Bias 5–10%; or LoA 10–15%), device-specific baseline strongly recommended; apparent changes ≤ 10% should not be interpreted as true change

x: **Cross-device comparison not recommended** (ICC < 0.50; Bias > 10%; or LoA > 15%), establish a new device-specific baseline before any longitudinal comparison; supplement with independent imaging modalities

All bias values are expressed as HRA minus comparator (positive = comparator underestimates; negative = comparator overestimates). LoA width = upper minus lower 95% limit of agreement (Bland–Altman). ICC = mean ICC(2,1) for that zone. For the 3-mm and 6-mm rings, ranges reflect variation across the four sectors (superior, nasal, inferior, temporal)

### Recommendations for longitudinal monitoring

The PIG has direct clinical implications that extend across multiple disease contexts:

The primary recommendation is to image patients on the same OCT device throughout their clinical course. When maintaining device consistency is achieved, the excellent within-device repeatability demonstrated here (ICC(3,1) > 0.85) ensures that apparent thickness changes reflect true anatomical change rather than device-related artifact [4, 5].

**If device switching is unavoidable**:

1. Establish a device-specific baseline on the new platform before comparing it to historical data.
2. Apply device-pair-specific calibration adjustments (e.g., expect ~ 14% lower central readings on TOPCON compared with HRA; apply bias-correction factors derived from the present data or published conversion models).
3. Use validated inter-device conversion models when available in the literature for the specific device combination.
4. Explicitly document the device model and software version at each imaging time point in the medical record.
5. For parafoveal and perifoveal monitoring, supplement OCT thickness measurements with complementary imaging modalities (e.g., fundus autofluorescence, microperimetry) when switching devices, to provide independent confirmation of structural status.

Location-specific interpretation thresholds should be applied. Apparent thickness changes of ≤ 10% in 3-mm and 6-mm ring sectors should be interpreted with caution when cross-device comparisons are involved, as they may fall within the device-related noise range identified in this study. High-stakes decisions (e.g., initiation or escalation of anti-VEGF therapy, diagnosis of drug-induced retinopathy) should prioritize serial measurements on the same device over exact absolute values from different platforms [7, 9].

### Limitations of the study

Several limitations should be acknowledged. First, participants were young, healthy adults (mean age 26.6 years), which maximizes image quality and segmentation accuracy but may not reflect the more complex imaging conditions encountered in older patients or in eyes with retinal pathology. This cohort design was intentionally adopted to establish the PIG under conditions that minimize confounders and yield the most reproducible measurements possible; however, the resulting PIG magnitudes should explicitly be interpreted as conservative lower bounds of inter-device disagreement in real-world clinical populations. In eyes with pathological structural changes, such as diabetic macular edema (DME), subretinal fluid, drusen, geographic atrophy, or epiretinal membranes, automated segmentation algorithms across different platforms are expected to diverge more substantially, as boundary ambiguity is compounded by disease-related tissue disruption. Prior reports in diseased eyes suggest that inter-device LoA can widen by a further 20–40% relative to healthy-eye benchmarks [7, 9]. Accordingly, the PIG gradients reported here define a baseline reference, and validation studies in clinical cohorts are warranted. Future research should prospectively characterize the PIG in DME, AMD, RVO, and myopic maculopathy populations, conditions in which parafoveal and perifoveal measurements are especially clinically consequential, and in older patient groups where image quality is typically lower. Such studies would also enable the derivation of disease-specific cross-device calibration equations and help define population-specific ETDRS zone thresholds for reliable inter-device comparison. In addition, refractive error was restricted ( > ± 1.0 D spherical equivalent excluded) to reduce confounding from axial-length–related magnification and retinal curvature differences that can disproportionately influence peripheral ETDRS sector measurements. As a result, the PIG magnitudes reported here may not generalize to high myopia, where increased axial length and peripheral geometric distortion can degrade segmentation stability and potentially amplify inter-device disagreement. Future studies should evaluate PIG behavior specifically in myopic and pathologic myopic cohorts. Second, automated proprietary segmentation was used without manual correction to reflect routine clinical practice, which may have introduced device-specific errors that careful manual review could partially reduce. Third, the sample size of *n* = 38, while consistent with comparable published cross-platform OCT agreement studies [9, 11] and analytically supported by the large total measurement count (> 8,100 discrete values) and high between-subject variance, does represent a limitation. The wide 95% confidence intervals on ICC estimates throughout the study reflect this constraint and should be noted when interpreting the precision of individual agreement metrics. The sample size precludes reliable stratified subgroup analyses by sex, age group, or axial length. Future studies with larger samples, enriched for specific patient populations such as older adults and eyes with DME, AMD, RVO, and myopic maculopathy, are needed to validate the PIG in diseased retinas, to derive formal inter-device conversion equations for clinical use, and to enable more precise sector-level agreement characterization. Fourth, although HRA Spectralis was used as the directional anchor for the primary bias analyses, it was not treated as a biological ground truth. To mitigate reference-device dependency, we performed supplementary all-pairwise cross-validation among all five OCT systems. Nevertheless, in the absence of a calibrated optical phantom or histological standard, absolute accuracy relative to true anatomical thickness cannot be established. While this is methodologically justified by its established research-grade status, active eye-tracking, and widespread use as a reference in prior cross-platform OCT literature [7, 9], it means that any systematic measurement offset intrinsic to HRA propagates uniformly through all reported bias estimates. A truly device-independent reference, such as a calibrated optical phantom or histological retinal measurement, would enable absolute rather than relative bias quantification, but is not feasible in a clinical in vivo study. Importantly, because all four comparator devices were assessed against the same HRA reference simultaneously, any fixed HRA-specific offset shifts all comparator biases by the same constant amount in the same direction. This structural property of the design ensures that the relative hierarchy of inter-device biases is mathematically invariant to reference choice and therefore does not represent an artifact of the reference selection. Fifth, while right–left eye differences were assessed via paired-samples t-tests, formal interocular correlation analysis was not performed; mild physiological interocular asymmetry may slightly attenuate ICC estimates in the bilateral analysis.

## Conclusion

OCT macular thickness measurements are internally consistent within individual devices but substantially non-interchangeable across platforms, particularly beyond the central macular subfield. The Peripheral Instability Gradient (PIG) is described as a progressive, device-independent deterioration in inter-device agreement from the central 1-mm ETDRS subfield (ICC up to 0.942) to the 6-mm perifoveal ring (ICC as low as 0.275–0.492) and represents a previously unquantified but clinically consequential source of measurement variability. This gradient was confirmed across all 10 pairwise device comparisons and was accompanied by systematic, directional biases: BMIZAR overestimated HRA by ~ 10.98% and TOPCON underestimated HRA by ~ 13.65%, with HUVITZ (+ 2.60%) and NIDEK (+ 3.64%) showing substantially smaller deviations (all expressed as HRA minus comparator). This study is the first to simultaneously characterize five contemporary OCT platforms, including a 400 kHz swept-source OCTA system (BMIZAR), within a unified framework explicitly designed to quantify the spatial gradient of cross-device agreement across ETDRS-defined macular regions. HUVITZ demonstrated the closest central agreement with HRA (ICC = 0.942; APE 2.39%), while NIDEK offered the most consistent performance across all ETDRS rings, making it the preferred comparator for peripheral macular monitoring.

For reliable longitudinal assessment, clinicians should prioritize device consistency throughout a patient’s clinical course. When device switching is unavoidable, device-specific baselines must be established on the new platform before comparing with historical data, bias-correction factors should be applied, and location-specific interpretation thresholds must account for the spatially structured noise described by the PIG. These evidence-based recommendations support more accurate clinical decision-making in the era of multi-platform OCT imaging.

## Supplementary Information

Below is the link to the electronic supplementary material.

Supplementary Material 1

## Data Availability

The datasets generated and/or analyzed during the current study are not publicly available due to institutional privacy policies, but are available from the corresponding author on reasonable request.
