# Supplementary material for: Peripheral instability gradient in macular thickness measurements across five optical coherence tomography systems: a prospective cross-sectional study of inter-device agreement and clinical monitoring risk
Source: BMC Med Imaging. 2026 May 30;26:374. doi: 10.1186/s12880-026-02480-3 (PMC13430785; doi:10.1186/s12880-026-02480-3)
Supplement: Supplementary file 1 — Supplementary Material 1 [file 12880_2026_2480_MOESM1_ESM.docx]

**Supplementary Table S1. All-pairwise cross-validation of macular thickness measurements among five OCT devices (eye-level analysis)**

| **Pair** | **ETDRS Region** | **n (eyes)** | **ICC(2,1)** | **Bias (A–B)** | **LoA Lower (µm)** | **LoA Upper (µm)** | **LoA Width (µm)** |
| --- | --- | --- | --- | --- | --- | --- | --- |
| BMIZAR–HRA | Central 1-mm | 76 | 0.47 | 32.11 | 7.51 | 56.71 | 49.2 |
| BMIZAR–HRA | 3-mm Superior | 76 | 0.45 | 24.14 | 4.47 | 43.81 | 39.35 |
| BMIZAR–HRA | 3-mm Nasal | 76 | 0.424 | 27.42 | 5.3 | 49.54 | 44.23 |
| BMIZAR–HRA | 3-mm Inferior | 76 | 0.434 | 25.42 | 7.29 | 43.54 | 36.25 |
| BMIZAR–HRA | 3-mm Temporal | 76 | 0.414 | 26.48 | 3.46 | 49.5 | 46.04 |
| BMIZAR–HRA | 6-mm Superior | 76 | 0.383 | 25.88 | 16.84 | 34.92 | 18.09 |
| BMIZAR–HRA | 6-mm Nasal | 76 | 0.381 | 27.91 | 13.99 | 41.82 | 27.83 |
| BMIZAR–HRA | 6-mm Inferior | 76 | 0.314 | 28.58 | 9.26 | 47.91 | 38.64 |
| BMIZAR–HRA | 6-mm Temporal | 76 | 0.439 | 22.08 | 5.72 | 38.45 | 32.72 |
| BMIZAR–HUVITZ | Central 1-mm | 76 | 0.369 | 38.67 | 11.91 | 65.42 | 53.51 |
| BMIZAR–HUVITZ | 3-mm Superior | 76 | 0.184 | 48.17 | 29.31 | 67.03 | 37.72 |
| BMIZAR–HUVITZ | 3-mm Nasal | 76 | 0.177 | 49.56 | 26.11 | 73.0 | 46.89 |
| BMIZAR–HUVITZ | 3-mm Inferior | 76 | 0.163 | 49.96 | 31.02 | 68.91 | 37.89 |
| BMIZAR–HUVITZ | 3-mm Temporal | 76 | 0.177 | 47.84 | 23.65 | 72.03 | 48.38 |
| BMIZAR–HUVITZ | 6-mm Superior | 76 | 0.116 | 53.31 | 39.6 | 67.01 | 27.41 |
| BMIZAR–HUVITZ | 6-mm Nasal | 76 | 0.157 | 50.94 | 32.81 | 69.06 | 36.25 |
| BMIZAR–HUVITZ | 6-mm Inferior | 76 | 0.095 | 57.29 | 33.42 | 81.16 | 47.75 |
| BMIZAR–HUVITZ | 6-mm Temporal | 76 | 0.11 | 53.18 | 29.6 | 76.75 | 47.14 |
| BMIZAR–NIDEK | Central 1-mm | 76 | 0.354 | 41.51 | 17.32 | 65.7 | 48.38 |
| BMIZAR–NIDEK | 3-mm Superior | 76 | 0.271 | 36.38 | 17.35 | 55.41 | 38.06 |
| BMIZAR–NIDEK | 3-mm Nasal | 76 | 0.275 | 39.89 | 19.93 | 59.84 | 39.91 |
| BMIZAR–NIDEK | 3-mm Inferior | 76 | 0.231 | 38.84 | 17.05 | 60.63 | 43.59 |
| BMIZAR–NIDEK | 3-mm Temporal | 76 | 0.237 | 40.39 | 16.4 | 64.39 | 47.98 |
| BMIZAR–NIDEK | 6-mm Superior | 76 | 0.144 | 42.82 | 25.48 | 60.15 | 34.68 |
| BMIZAR–NIDEK | 6-mm Nasal | 76 | 0.209 | 40.16 | 21.31 | 59.0 | 37.69 |
| BMIZAR–NIDEK | 6-mm Inferior | 76 | 0.128 | 44.39 | 15.36 | 73.41 | 58.05 |
| BMIZAR–NIDEK | 6-mm Temporal | 76 | 0.145 | 41.96 | 20.22 | 63.69 | 43.47 |
| BMIZAR–TOPCON | Central 1-mm | 76 | 0.202 | 62.88 | 37.96 | 87.8 | 49.84 |
| BMIZAR–TOPCON | 3-mm Superior | 76 | 0.131 | 54.8 | 32.02 | 77.59 | 45.56 |
| BMIZAR–TOPCON | 3-mm Nasal | 76 | 0.143 | 58.8 | 34.98 | 82.63 | 47.65 |
| BMIZAR–TOPCON | 3-mm Inferior | 76 | 0.139 | 56.17 | 36.51 | 75.83 | 39.32 |
| BMIZAR–TOPCON | 3-mm Temporal | 76 | 0.139 | 56.63 | 32.59 | 80.68 | 48.09 |
| BMIZAR–TOPCON | 6-mm Superior | 76 | 0.123 | 53.44 | 43.5 | 63.38 | 19.89 |
| BMIZAR–TOPCON | 6-mm Nasal | 76 | 0.128 | 56.28 | 38.81 | 73.74 | 34.93 |
| BMIZAR–TOPCON | 6-mm Inferior | 76 | 0.111 | 55.66 | 34.94 | 76.38 | 41.44 |
| BMIZAR–TOPCON | 6-mm Temporal | 76 | 0.135 | 49.39 | 28.55 | 70.23 | 41.68 |
| HRA–HUVITZ | Central 1-mm | 76 | 0.936 | 6.56 | -2.94 | 16.06 | 19.0 |
| HRA–HUVITZ | 3-mm Superior | 76 | 0.468 | 24.03 | 10.96 | 37.1 | 26.15 |
| HRA–HUVITZ | 3-mm Nasal | 76 | 0.537 | 22.14 | 9.56 | 34.71 | 25.15 |
| HRA–HUVITZ | 3-mm Inferior | 76 | 0.454 | 24.55 | 14.97 | 34.13 | 19.16 |
| HRA–HUVITZ | 3-mm Temporal | 76 | 0.524 | 21.36 | 11.78 | 30.95 | 19.16 |
| HRA–HUVITZ | 6-mm Superior | 76 | 0.336 | 27.43 | 15.66 | 39.19 | 23.53 |
| HRA–HUVITZ | 6-mm Nasal | 76 | 0.433 | 23.03 | 6.01 | 40.05 | 34.03 |
| HRA–HUVITZ | 6-mm Inferior | 76 | 0.301 | 28.71 | 15.95 | 41.46 | 25.51 |
| HRA–HUVITZ | 6-mm Temporal | 76 | 0.262 | 31.09 | 12.36 | 49.82 | 37.45 |
| HRA–NIDEK | Central 1-mm | 76 | 0.894 | 9.4 | -0.95 | 19.75 | 20.7 |
| HRA–NIDEK | 3-mm Superior | 76 | 0.705 | 12.24 | -3.51 | 28.0 | 31.51 |
| HRA–NIDEK | 3-mm Nasal | 76 | 0.756 | 12.46 | -1.91 | 26.84 | 28.75 |
| HRA–NIDEK | 3-mm Inferior | 76 | 0.665 | 13.43 | -2.76 | 29.61 | 32.38 |
| HRA–NIDEK | 3-mm Temporal | 76 | 0.7 | 13.92 | 1.82 | 26.01 | 24.19 |
| HRA–NIDEK | 6-mm Superior | 76 | 0.489 | 16.93 | 0.77 | 33.1 | 32.33 |
| HRA–NIDEK | 6-mm Nasal | 76 | 0.65 | 12.25 | -4.92 | 29.42 | 34.34 |
| HRA–NIDEK | 6-mm Inferior | 76 | 0.475 | 15.8 | -5.35 | 36.95 | 42.3 |
| HRA–NIDEK | 6-mm Temporal | 76 | 0.39 | 19.87 | 1.51 | 38.23 | 36.72 |
| HRA–TOPCON | Central 1-mm | 76 | 0.508 | 30.77 | 21.19 | 40.35 | 19.16 |
| HRA–TOPCON | 3-mm Superior | 76 | 0.319 | 30.66 | 12.65 | 48.68 | 36.04 |
| HRA–TOPCON | 3-mm Nasal | 76 | 0.388 | 31.38 | 17.9 | 44.86 | 26.96 |
| HRA–TOPCON | 3-mm Inferior | 76 | 0.355 | 30.75 | 19.68 | 41.83 | 22.14 |
| HRA–TOPCON | 3-mm Temporal | 76 | 0.359 | 30.15 | 17.87 | 42.44 | 24.58 |
| HRA–TOPCON | 6-mm Superior | 76 | 0.348 | 27.56 | 18.88 | 36.23 | 17.35 |
| HRA–TOPCON | 6-mm Nasal | 76 | 0.335 | 28.37 | 12.1 | 44.63 | 32.53 |
| HRA–TOPCON | 6-mm Inferior | 76 | 0.345 | 27.07 | 18.1 | 36.05 | 17.94 |
| HRA–TOPCON | 6-mm Temporal | 76 | 0.33 | 27.31 | 11.27 | 43.34 | 32.07 |
| HUVITZ–NIDEK | Central 1-mm | 76 | 0.951 | 2.84 | -9.54 | 15.23 | 24.77 |
| HUVITZ–NIDEK | 3-mm Superior | 76 | 0.717 | -11.79 | -26.62 | 3.04 | 29.66 |
| HUVITZ–NIDEK | 3-mm Nasal | 76 | 0.763 | -9.67 | -27.04 | 7.7 | 34.74 |
| HUVITZ–NIDEK | 3-mm Inferior | 76 | 0.684 | -11.12 | -28.45 | 6.2 | 34.65 |
| HUVITZ–NIDEK | 3-mm Temporal | 76 | 0.82 | -7.45 | -21.21 | 6.31 | 27.52 |
| HUVITZ–NIDEK | 6-mm Superior | 76 | 0.64 | -10.49 | -26.76 | 5.77 | 32.53 |
| HUVITZ–NIDEK | 6-mm Nasal | 76 | 0.661 | -10.78 | -30.42 | 8.85 | 39.27 |
| HUVITZ–NIDEK | 6-mm Inferior | 76 | 0.499 | -12.9 | -36.02 | 10.22 | 46.24 |
| HUVITZ–NIDEK | 6-mm Temporal | 76 | 0.471 | -11.22 | -35.58 | 13.14 | 48.72 |
| HUVITZ–TOPCON | Central 1-mm | 76 | 0.606 | 24.21 | 12.87 | 35.56 | 22.69 |
| HUVITZ–TOPCON | 3-mm Superior | 76 | 0.776 | 6.63 | -11.75 | 25.02 | 36.77 |
| HUVITZ–TOPCON | 3-mm Nasal | 76 | 0.78 | 9.25 | -7.41 | 25.9 | 33.31 |
| HUVITZ–TOPCON | 3-mm Inferior | 76 | 0.869 | 6.21 | -5.52 | 17.93 | 23.46 |
| HUVITZ–TOPCON | 3-mm Temporal | 76 | 0.793 | 8.79 | -4.47 | 22.05 | 26.53 |
| HUVITZ–TOPCON | 6-mm Superior | 76 | 0.903 | 0.13 | -12.35 | 12.62 | 24.97 |
| HUVITZ–TOPCON | 6-mm Nasal | 76 | 0.722 | 5.34 | -16.85 | 27.52 | 44.37 |
| HUVITZ–TOPCON | 6-mm Inferior | 76 | 0.887 | -1.63 | -14.76 | 11.49 | 26.25 |
| HUVITZ–TOPCON | 6-mm Temporal | 76 | 0.686 | -3.79 | -26.98 | 19.41 | 46.38 |
| NIDEK–TOPCON | Central 1-mm | 76 | 0.652 | 21.37 | 7.6 | 35.14 | 27.54 |
| NIDEK–TOPCON | 3-mm Superior | 76 | 0.483 | 18.42 | -1.93 | 38.78 | 40.71 |
| NIDEK–TOPCON | 3-mm Nasal | 76 | 0.564 | 18.92 | 0.04 | 37.8 | 37.76 |
| NIDEK–TOPCON | 3-mm Inferior | 76 | 0.528 | 17.33 | -1.9 | 36.56 | 38.47 |
| NIDEK–TOPCON | 3-mm Temporal | 76 | 0.583 | 16.24 | -0.47 | 32.95 | 33.42 |
| NIDEK–TOPCON | 6-mm Superior | 76 | 0.644 | 10.62 | -5.26 | 26.51 | 31.77 |
| NIDEK–TOPCON | 6-mm Nasal | 76 | 0.496 | 16.12 | -5.26 | 37.5 | 42.76 |
| NIDEK–TOPCON | 6-mm Inferior | 76 | 0.563 | 11.27 | -10.52 | 33.06 | 43.58 |
| NIDEK–TOPCON | 6-mm Temporal | 76 | 0.608 | 7.43 | -14.48 | 29.35 | 43.83 |
| ICC: Intraclass Correlation Coefficient, ICC(2,1), two-way random-effects model, absolute agreement, single measurement. Bias = Device A − Device B (µm). LoA_L and LoA_U: lower and upper 95% limits of agreement. LoA_width = LoA_U − LoA_L. Analysis performed at eye level; three repeated scans per eye were averaged before comparison (n = 76 eyes).  Unlike the main HRA-anchored Bland–Altman table, this supplementary table reports absolute differences in micrometers for each pairwise comparison | | | | | | | |
